# From *Carica papaya* Waste to Car Door Panels: Alkaline Extraction Effects on Polyester Nonwoven Composites

**DOI:** 10.3390/polym18151891

**Published:** 2026-07-31

**Authors:** Abel Emmanuel Njom, Jean Jalin Eyinga Biwôlé, Armel Edwige Mewoli, Marie Josette Ndengue, Roland Yves Olembe, Cesar Segovia, Fabien Betene Ebanda, Florent Biyeme, Christian Lidam Dara, Aron Désiré Nyana Ko’o, Meva’a Lucien, Mohammad Sadeghi, Pierre Girods, Antonio Pizzi, Atangana Ateba, Nikolaos A. Papadopoulos, Antonios N. Papadopoulos

**Affiliations:** 1 Department of Mechanics, Materials, Manufacturing and Artificial Intelligence, University of Bertoua, Bertoua P.O. Box 416, Cameroon; abelkamen@gmail.com; 2 Laboratory of Mechanics, Department of Mechanical Engineering, Higher Technical Teacher Training College (HTTTC), University of Douala, Douala P.O. Box 1872, Cameroon; marielongo84@gmail.com (M.J.N.); fabetene@yahoo.fr (F.B.E.); lidamchristian@gmail.com (C.L.D.); nyanakooarondesire@gmail.com (A.D.N.K.); atebaatanagana1986@gmail.com (A.A.); 3 Laboratory of Forest Resources and Wood Valorization, University of Douala, Douala P.O. Box 2701, Cameroon; mewoliarmel@yahoo.fr; 4 Department of Industrial and Mechanical Engineering, National Advanced School of Engineering, University of Yaoundé I, Yaoundé P.O. Box 8390, Cameroon; rolandolembe@gmail.com (R.Y.O.); florentbiyeme@gmail.com (F.B.); jrl67_mevaa@yahoo.com (M.L.); 5 Laboratory of Materials Mechanics, Structures and Integrated Manufacturing, National Advanced School of Engineering, University of Yaoundé I, Yaoundé P.O. Box 8390, Cameroon; 6 Laboratory for Studies and Research on Wood Material (LERMAB), National Graduate School of Wood Science and Engineering (ENSTIB), Université de Lorraine, 27 rue Philippe Seguin, BP 1041, 88051 Épinal, France; cesar.segovia@univ-lorraine.fr (C.S.); mohammad.sadeghisadeghabad@univ-lorraine.fr (M.S.); pierre.girods@univ-lorraine.fr (P.G.); antonio.pizzi@univ-lorraine.fr (A.P.); 7 Department of Materials Science and Engineering, University of Ioannina, 45110 Ioannina, Greece; 8 Laboratory of Wood Science-Chemistry & Technology, Department of Natural Environment & Climate Resilience, Democritus University of Thrace, 1 km Drama-Mikrochoriou, 66100 Drama, Greece

**Keywords:** *Carica papaya*, natural fibers, alkaline treatment, nonwoven composites, unsaturated polyester, mechanical properties, thermal stability, low-velocity impact

## Abstract

The growing interest in sustainable materials has stimulated research into bio-based reinforcements. This study investigates the influence of fiber extraction methods on the properties of *Carica papaya* (CP) pseudostem fibers and on the performance of polyester-based nonwoven composites. Fibers were extracted by water retting (CPFR) and alkaline treatments using 5 wt.% NaOH (CPF5) and 10 wt.% NaOH (CPF10). Preliminary characterization confirmed the suitability of CP fibers as reinforcements for nonwoven composite materials. After carding, the fiber mats were impregnated with unsaturated polyester resin (UP), consolidated by compression molding, and post-cured in an oven. The experimental results showed that alkaline extraction significantly improved fiber–matrix adhesion and reduced water uptake. CPF10UP exhibited the lowest porosity (12.7%) and water absorption (2.62%). CPF5UP provided the best overall mechanical performance, achieving the highest tensile and flexural properties compared with neat polyester resin. In contrast, CPF10UP maximized tensile stiffness, impact resistance, and thermal stability. Radar chart analysis revealed two distinct optimization strategies: CPF5UP for superior mechanical performance and CPF10UP for enhanced moisture resistance and impact durability. Overall, the 5 wt.% NaOH treatment provided the best compromise between strength and stiffness, whereas the 10 wt.% NaOH treatment favored higher rigidity, thermal stability, and water resistance. Derived from agricultural waste, CP fibers demonstrate strong potential for semi-structural automotive interior components, particularly door panels, while supporting circular economy principles and sustainable material development.

## 1. Introduction

Agricultural and agro-industrial activities continuously generate increasing amounts of by-products during both harvesting and processing operations [1,2]. When improperly managed, these residuals can become major sources of environmental pollution and economic losses. In this context, the emergence of the circular economy concept and the growing demand for sustainable development have encouraged the valorization of agricultural residues into high-value-added materials, thereby promoting more efficient and sustainable management of natural resources [1,3].

Among the most promising approaches, several studies [2,4] have demonstrated that agricultural residuals, such as tomato and maize stalks, can be transformed into technical fibers for the production of biodegradable composites with bio-based matrices. These investigations have shown that the conversion of agro-industrial by-products into engineered fibrous reinforcements, combined with suitable polymer matrices, can lead to materials exhibiting satisfactory mechanical performance while fitting within a circular economy framework. Consequently, the incorporation of agricultural waste into recyclable or compostable materials offers new opportunities for the eco-design of products with reduced environmental impact.

Within this transition toward a circular bioeconomy, lignocellulosic resources derived from plant biomass, particularly natural fibers, have attracted considerable attention. Owing to their wide availability, low density, biodegradability, and low cost, natural fibers are increasingly used as reinforcements in composite materials, especially thermosetting polymers such as unsaturated polyester (UP) resins. Unsaturated polyester matrices are among the most widely used thermosetting systems in composite manufacturing due to their good mechanical performance, dimensional stability, ease of processing, and industrial relevance. Their utilization follows a dual objective: improving material performance while reducing environmental impacts [1,3].

Natural fibers obtained from agricultural by-products represent a sustainable alternative to energy-intensive synthetic fibers such as glass and carbon fibers, whose production is associated with significant energy consumption and emissions of volatile organic compounds [5]. Lightweight, renewable, and biodegradable, these lignocellulosic fibers offer attractive specific mechanical properties, high specific stiffness, vibration-damping capability, and thermal and acoustic insulation performance [6,7,8,9]. These advantages make them suitable candidates for a wide range of industrial applications, including aerospace, automotive, sports equipment, marine structures, and construction materials [5,6,10,11]. In particular, automotive interior components have become a major application field for natural fiber composites because they require a balance between lightweight design, mechanical performance, acoustic properties, and sustainability.

In the search for new natural fiber sources for automotive applications, tropical agricultural residuals represent a particularly promising resource. Cameroon, for example, produces approximately 43,000 tons of papaya annually [12]. This crop generates substantial quantities of lignocellulosic biomass from *Carica papaya* (CP) pseudostems, which remain largely unexploited after the fruit production cycle. Unlike lignified trees, the papaya stem consists of a network of fibrovascular bundles embedded within a soft parenchymatous matrix, giving rise to fibers with distinctive mechanical characteristics [13]. Beyond their technical interest, these fibers also present significant economic potential because the raw material is an abundant agricultural residual generally discarded after fruit harvesting, resulting in low procurement costs and creating opportunities for local value addition. The valorization of these residues therefore represents an attractive strategy for simultaneously reducing environmental burdens, creating additional income for farmers, and supplying renewable raw materials for the composite industry.

Several studies conducted in India, Brazil, and Cameroon support this potential. Papaya fibers contain relatively high cellulose contents (38–62%), exhibit crystallinity indices reaching 56.3%, and possess low densities ranging from 0.94 to 1.12 g·cm^−3^ [14,15,16]. Saravana Kumaar et al. [14] reported a cellulose content of 38.7%, a crystallinity index of 56.3%, and a density of 0.943 g·cm^−3^. Kempe et al. [15] highlighted a multiscale cellular architecture responsible for a flexural modulus of approximately 2.5 GPa. Lautenschläger et al. [16] reported a tensile strength of 100 MPa and a Young’s modulus of 10 GPa. More recently, Ze et al. [17] obtained fiber mats exhibiting a modulus of 8.73 GPa, a tensile strength of 58.98 MPa, and a flexural strength of 920 MPa. Parfait et al. [18] demonstrated that alkaline treatment using NaOH improves compatibility with polyester matrices, leading to reduced density and enhanced mechanical performance. Similarly, Mono et al. [19] reported that alkaline treatment increases fiber crystallinity, removes amorphous surface impurities, and enhances fiber–matrix interfacial adhesion.

Although alkaline treatment is a well-established strategy for improving natural fiber performance, the objective of the present study is not to claim a new chemical modification route. Instead, the scientific contribution lies in understanding how different extraction conditions (water retting and NaOH treatments at 5 wt.% and 10 wt.%), combined with carded nonwoven reinforcement architecture and UP processing, influence the structure–property relationships of the resulting composites.

Despite these encouraging findings, most published studies have focused on the characterization of individual papaya fibers or randomly oriented fiber systems, whereas the development of carded nonwoven reinforcements for structural thermosetting composites remains unexplored. Furthermore, the combined influence of fiber extraction route, carding process, compression molding, and post-curing on the physical, mechanical, thermal, and impact performance of *Carica papaya*/UP composites has not yet been systematically investigated. Therefore, the following research question arises: can carded nonwoven mats produced from *Carica papaya* pseudostem fibers serve as effective reinforcements for unsaturated polyester biocomposites while meeting the technical and environmental requirements of the automotive industry?

To address this question, CP fibers were extracted, carded, and incorporated into unsaturated polyester (UP) composites. The UP resin was selected as the polymer matrix because of its extensive use in industrial composite applications and its compatibility with compression molding processes commonly employed for automotive components. In addition to fiber characteristics, the manufacturing route is a critical factor governing composite performance because it directly influences resin impregnation, void content, interfacial bonding, and curing efficiency. Unlike conventional cold molding, which requires approximately 24 h for demolding and often results in incomplete curing [20], and unlike thermocompression alone, which provides limited control over porosity, the present study combines compression molding with oven post-curing. This coupled process removes excess resin, reduces porosity, and promotes complete polymerization within a short processing time while preserving the thermal integrity of the fibers [20,21,22].

To the best of our knowledge, this is the first study investigating the use of carded nonwoven mats derived from *Carica papaya* pseudostem fibers as reinforcements for unsaturated polyester composites manufactured by compression molding followed by oven post-curing for automotive interior applications.

The resulting biocomposites were manufactured through compression molding followed by oven post-curing, and their properties were evaluated and compared with those of conventional composite materials. The novelty of this work is therefore related to the development of a complete composite processing strategy combining agricultural residue valorization, controlled fiber extraction, nonwoven reinforcement design, and polymer processing optimization. This approach provides new insights into the interaction between lignocellulosic fiber characteristics, UP matrix behavior, processing parameters, and final composite performance, which is fully aligned with the scope of polymer science and engineering.

To assess this potential, three fiber extraction routes, including water retting and two alkaline treatments (5 wt.% and 10 wt.% NaOH), were investigated. Carded nonwoven mats were subsequently produced and incorporated into an unsaturated polyester matrix through compression molding followed by oven post-curing. The physical, mechanical, thermal, and low-velocity impact properties of the resulting composites were characterized to identify the optimum extraction treatment and evaluate their suitability for lightweight automotive interior components, particularly door panels, where mechanical performance, thermal stability, impact resistance, and sustainability are simultaneously required.

## 2. Material and Methods

### 2.1. Raw Material and Extraction of Carica papaya Fibers

**Figure 1:**
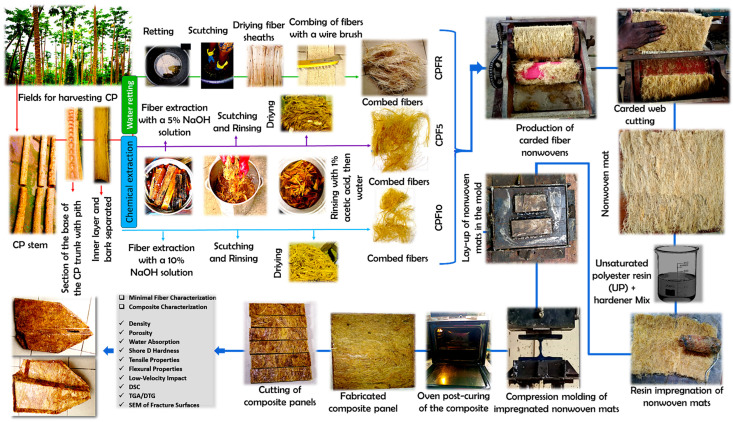
Processing steps from *Carica papaya* pseudostem waste to fiber-reinforced polyester composites.

*Carica papaya* (CP) pseudostems were collected from plantations located in Njombe (4°34′60″ N et 9°39′0″ E), Littoral Region, Cameroon, after the completion of the fruit production cycle. The stems were cut approximately 50 cm above ground level, and the outer bark was manually separated from the inner tissues for fiber extraction (Figure 1).

Three extraction methods were investigated: water retting, 5 wt.% sodium hydroxide (NaOH) treatment, and 10 wt.% NaOH treatment. For water retting, the bark strips were immersed in stagnant water for five weeks under the natural climatic conditions of Douala, Cameroon, where the ambient temperature ranged from 27 to 30 °C and the relative humidity ranged from 80 to 85%, to promote the biological degradation of non-fibrous constituents. After retting, the fibers were manually separated, washed with tap water, rinsed with distilled water, and air-dried in the shade for one week.

For alkaline extraction, the bark strips were immersed in 5 wt.% and 10 wt.% NaOH solutions for 2 h using a solid-to-liquid ratio of 1:20. After treatment, the fibers were thoroughly rinsed with deionized water, neutralized in a 1 wt.% acetic acid solution, and washed again with distilled water to remove residual alkali. The fibers were then oven-dried until a constant weight was reached.

Fibers obtained by water retting, 5 wt.% NaOH treatment, and 10 wt.% NaOH treatment were designated as CPFR, CPF5, and CPF10, respectively. The fiber extraction yield was calculated as the ratio of the dry mass of extracted fibers to the initial dry mass of CP bark according to Equation (1):

(1) $$R(\%)=\frac{m_{f}}{m_{i}}\times 100$$

where $m_{f}$ is the dry mass of extracted fibers (g) and $m_{i}$ is the initial dry mass of CP bark (g).

### 2.2. Fiber Characterization

A preliminary characterization was conducted to establish relationships between fiber properties and the performance of the resulting composites.

Fiber surface morphology was examined using scanning electron microscopy (SEM). Prior to observation, the samples were mounted on conductive holders and sputter-coated with a thin conductive layer. SEM micrographs were used to assess the effects of the extraction method on surface cleanliness, impurity removal, fibrillation, and fiber bundle individualization.

The average fiber diameter was measured by optical microscopy from multiple measurements taken along each fiber. The acquired images were analyzed using ImageJ software. Fiber length distribution after carding was determined by individually dispersing the fibers on the glass surface of a Canon scanner to avoid overlap. The samples were scanned, and six images were acquired for each condition. Between 150 and 200 fibers were measured using ImageJ software. These measurements were used to evaluate the influence of the extraction process on fiber length preservation and suitability for nonwoven mat production.

The chemical composition of the fibers, including cellulose, hemicelluloses, lignin, pectins, waxes, and extractives, was determined according to TAPPI standard methods [13]. Functional group evolution was analyzed by attenuated total reflectance Fourier-transform infrared spectroscopy (ATR–FTIR) using a Shimadzu IRAffinity-1 CE spectrometer (Kyoto, Japan) in the 400–4000 cm^−1^ range. The characteristic absorption bands were used to monitor the removal of amorphous constituents, particularly pectins, hemicelluloses, and part of the lignin, following alkaline extraction.

Fiber density was determined using a pycnometer method. Moisture content was measured from the mass difference before and after oven drying to constant weight. Water absorption was evaluated after 24 h immersion in distilled water and calculated using Equation (2):

(2) $$A(\%)=\frac{m_{h}-m_{s}}{m_{s}}\times 100$$

where $m_{s}$ is the initial dry mass of the sample (g) and $m_{h}$ is the mass after 24 h immersion (g).

The mechanical properties of individual fibers were determined by uniaxial tensile testing according to ASTM D3379-75 [23]. Fibers were mounted on paper frames to ensure proper alignment and prevent prestressing prior to testing. Tensile tests were carried out on an LDW-1 universal testing machine equipped with a 1 kN load cell at a crosshead speed of 1 mm min^−1^. Tensile strength, Young’s modulus, and elongation at break were determined from the resulting stress–strain curves.

### 2.3. Manufacture of Nonwoven Mats

The different processing steps involved in transforming *Carica papaya* pseudostems into fibers, nonwoven mats, and composite materials are illustrated in Figure 1. Following extraction by either water retting or alkaline treatment, the fibers were manually opened using a metallic brush equipped with steel pins at a density of 5 pins cm^−2^. This operation disrupted the natural honeycomb-like structure of the fibers and promoted the separation of individual fiber bundles.

The fibers were subsequently carded using a laboratory-scale carding machine equipped with two metallic-toothed rollers having a tooth density of 150 teeth cm^−2^ and operating at 20 rpm. Carding promoted preferential fiber alignment along the machine direction (MD), which was subsequently investigated throughout this study. Owing to the relatively high stiffness of CP fibers and the simplified two-roller configuration of the carding machine, three successive carding passes were required to obtain a homogeneous fiber web.

The resulting web was released from the main roller through lateral cutting, manually removed, and trimmed to the desired dimensions. Regions exhibiting nonuniform fiber distribution were eliminated using a light transmission test performed on a backlit transparent Plexiglas plate.

The fiber length distribution after carding was determined as described in Section 2.2. The physical characteristics of the nonwoven mats were evaluated through areal density and thickness measurements. Areal density was calculated from the ratio between mat mass and surface area, while thickness was measured at several locations using a digital caliper.

Homogeneous nonwoven mats produced from CPFR, CPF5, and CPF10 fibers were cut using a cardboard template matching the dimensions of the composite mold (210 × 150 mm^2^). The resulting mats exhibited areal densities of 501.37, 598.39, and 529.24 g m^−2^ and average thicknesses of approximately 9.0 ± 1.0, 11.0 ± 1.0, and 9.5 ± 1.5 mm, respectively.

### 2.4. Composite Manufacturing Process

Unsaturated polyester (UP) resin was mixed with 3 wt.% peroxide curing agents in the laboratory. Each nonwoven layer was manually impregnated using a metallic roller to facilitate resin penetration throughout the fibrous structure.

The manufacturing route plays a crucial role in determining the final composite performance. Unlike conventional cold molding, which requires approximately 24 h before demolding and often results in incomplete curing [20], and unlike thermocompression alone, which provides limited control over porosity, a two-step process combining compression molding and oven post-curing was adopted. During molding, the impregnated mats were placed between two aluminum release films and introduced into a custom-designed welded steel mold equipped with demolding grooves. A pressure of 4 MPa was applied for 10 min to remove excess resin, reduce composite weight, and minimize air entrapment. Metallic spacers with a thickness of 4 mm were positioned at the mold edges to ensure a constant composite thickness throughout the panel. Following compression molding, the laminates were post-cured in a laboratory oven at 180 °C for 30 min. This temperature remained below the thermal degradation threshold of the fibers while significantly accelerating crosslinking and ensuring complete polymerization compared with conventional cold curing [20]. The combination of pressure and thermal post-curing therefore provides an effective compromise between weight reduction, porosity control, processing time, and preservation of fiber integrity. A schematic representation of the manufacturing process is shown in Figure 1.

The resulting composite panels were machined according to the relevant ASTM standards. Tensile specimens (125 × 12.5 mm^2^) were prepared following ASTM D3039, while three-point bending specimens (127 × 12.7 mm^2^) were prepared according to ASTM D790. Density and water absorption measurements were performed on prismatic specimens measuring 25 × 25 mm^2^.

To evaluate the anisotropy induced by the carding process, the composites were tested in two principal directions: the machine direction (MD), corresponding to the preferential fiber alignment direction, and the cross direction (CD). This approach enabled assessment of the influence of fiber orientation on the mechanical behavior of the composites.

The composites were designated according to the fiber extraction method employed. The neat polyester resin was designated UP. Composites reinforced with fibers obtained by water retting, 5 wt.% NaOH treatment, and 10 wt.% NaOH treatment were designated CPFRUP, CPF5UP, and CPF10UP, respectively. The constituent weight fractions are summarized in Table 1.

### 2.5. Physical Characterization of the Composites

The density of the manufactured composites was determined using two complementary methods: the geometric method and the hydrostatic method. Geometric density was calculated from the mass and apparent volume of the specimens; the latter being determined from their dimensions according to NF P 94-054. Hydrostatic density was determined by immersion in 96 vol.% ethanol, used as the reference liquid.

The open porosity of the composites was estimated from the difference between geometric and hydrostatic densities according to Equation (3):

(3) $$P(\%)=\left(1-\frac{\rho_{g}}{\rho_{h}}\right)\times 100$$

where $\rho_{g}$ is the geometric density (g cm^−3^) and $\rho_{h}$ is the hydrostatic density (g cm^−3^).

Water absorption was evaluated according to the principles of ASTM D570. Prior to testing, the specimens were oven-dried at 60 °C until constant weight was reached and subsequently immersed in distilled water at room temperature. At predetermined intervals, the specimens were removed, gently wiped with absorbent paper to eliminate surface water, and weighed using a balance with an accuracy of 10^−3^ g.

The water absorption rate was calculated according to Equation (4):

(4) $$Abs\;(\%)=\frac{{W(t)}_{f}-{W(t)}_{i}}{{W(t)}_{i}}\times 100$$

where ${W(t)}_{f}$ is the mass of the specimen at time (t) and ${W(t)}_{i}$ is its initial dry mass.

### 2.6. Mechanical Characterization of the Composites

***Tensile testing.*** Tensile tests were performed in accordance with ASTM D3039 using a universal testing machine (LDW-1, China) equipped with a 10 kN load cell. Composite specimens were machined along both the machine direction (MD) and the cross direction (CD) of the nonwoven mats, with nominal dimensions of 125 × 12.5 × 4.3 mm^3^**.** The tests were conducted at a constant crosshead speed of 2 mm min^−1^. The tensile modulus, ultimate tensile strength, and elongation at break were determined from the stress–strain curves. Six specimens were tested for each composite formulation, and the reported values correspond to the mean ± standard deviation.

***Three-point bending test.*** Flexural tests were performed according to ASTM D790 using the same universal testing machine (LDW-1, China). The specimens measured 127 × 12.7 × 4.3 mm^3^ and were tested with a span-to-thickness ratio of 16:1. The crosshead speed was maintained at 2 mm min^−1^. Specimens were cut along both the MD and CD directions to assess the anisotropy induced by preferential fiber orientation during carding. The flexural modulus (*Ef*) was calculated from the linear portion of the stress–strain curve between 0.05% and 0.25% strain. Six specimens were tested for each formulation.

***Shore D hardness test.*** Surface hardness was measured using a digital Shore D durometer according to ASTM D2240 [24]. Five specimens were tested for each formulation, and five indentations were performed at different locations on each specimen, resulting in a total of 25 measurements per formulation. The reported values correspond to the mean hardness value ± standard deviation.

**Figure 2:**
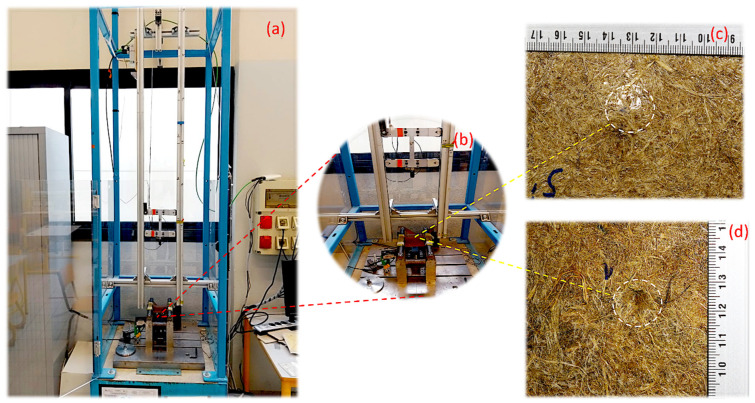
Experimental setup for the low-velocity impact (LVI) test: (**a**) low-velocity impact testing machine; (**b**) specimen mounted in the impact fixture before testing; (**c**) impacted specimen showing damage on the convex face; and (**d**) impacted specimen showing damage on the concave face.

***Low-velocity impact (LVI) Test.*** Low-velocity impact tests (Figure 2) were carried out using an instrumented drop-weight tower equipped with a hemispherical impactor of 16 mm diameter [25]. Composite specimens measuring 150 × 100 × 4.3 mm^3^ were tested under a simply supported configuration using an impact mass of 850 g. Force and displacement signals were continuously recorded to determine the maximum impact force, maximum displacement, and absorbed energy. The results presented in this study correspond to tests performed at a drop height of 0.50 m, corresponding to an initial impact energy of approximately 4.17 J. This condition provided the most reliable basis for comparing the different composite formulations.

### 2.7. Thermal Analysis of the Composites

The thermal behavior of the composites was investigated using differential scanning calorimetry (DSC) and thermogravimetric analysis (TGA).

***Differential Scanning Calorimetry (DSC).*** DSC measurements were carried out using a NETZSCH DSC 404 F1 analyzer (NETZSCH-Gerätebau GmbH, Selb, Germany). Approximately 20 mg of each sample was placed in an aluminum crucible and heated from 24 °C to 500 °C at a rate of 10 K min^−1^ under a nitrogen atmosphere. These analyses were used to identify thermal transitions associated with the polyester matrix and residual lignocellulosic constituents.

***Thermogravimetric analysis (TGA)***. Thermogravimetric analyses were performed using a NETZSCH STA 449 F3 Jupiter thermal analyzer (NETZSCH-Gerätebau GmbH, Selb, Germany). Samples weighing approximately 30 ± 1 mg were heated from 20 °C to 600 °C at a rate of 20 K min^−1^ under nitrogen atmosphere. The tests provided characteristic degradation temperatures, mass loss profiles, and residual mass contents.

### 2.8. Morphological Observation of Fracture Surfaces

Scanning electron microscopy (SEM). The fracture surfaces of the composites after mechanical testing were examined using a Carl Zeiss Supra 25 scanning electron microscope (SEM). Prior to observation, the fractured specimens were dried under ambient laboratory conditions and mounted on aluminum SEM stubs using double-sided conductive carbon tape. To prevent electrostatic charging during imaging, the samples were coated with a thin conductive aluminum layer by vacuum sputter deposition using a sputter coater. The aluminum coating, with an approximate thickness of 10–15 nm, was deposited under low-pressure conditions to ensure a uniform conductive surface without altering the fracture morphology. SEM observations were carried out at an accelerating voltage of 20 kV, with working distances adjusted between 8 and 12 mm depending on the magnification. Micrographs were acquired at magnifications ranging from ×50 to ×1500 in order to characterize the fracture morphology at different scales. These observations were used to identify the dominant damage mechanisms, including fiber fracture, fiber pull-out, fiber-matrix debonding, matrix cracking, and the presence of residual voids.

### 2.9. Statistical Analysis

Experimental results for the mechanical and hardness properties are reported as mean ± standard deviation. One-way analysis of variance (ANOVA) was performed to evaluate the effect of composite formulation on the investigated properties. When significant differences were detected, Tukey’s honestly significant difference (HSD) post-hoc test was applied to identify statistically distinct groups. Statistical significance was established at (*p* < 0.05).

## 3. Results and Discussion

### 3.1. Fiber Extraction Yield and Preliminary Characterization

Although the extraction yields of *Carica papaya* (CP) fibers obtained in this study were relatively low (10.79–13.10%), these values should be considered in relation to the origin of the raw material. CP fibers are extracted from pseudostem residues generated after fruit harvesting, providing an opportunity to valorize agricultural waste without competing with food production. The conversion of such lignocellulosic residues into reinforcement materials represents a sustainable approach consistent with circular bioeconomy strategies [3,4,5].

The reduction in fiber yield after alkaline treatment was mainly associated with the removal of non-cellulosic constituents, including pectins, hemicelluloses, waxes, and part of lignin. However, a lower yield does not necessarily indicate poor suitability for composite applications, since the removal of amorphous components can improve cellulose enrichment, surface properties, and fiber–matrix compatibility [9]. Previous studies on *Carica papaya* fibers have confirmed that extraction methods strongly influence their chemical composition and reinforcement potential in biocomposites [13,16,19].

Therefore, the feasibility of CP fibers as polyester composite reinforcement should be evaluated based on their overall performance rather than extraction yield alone. Despite the moderate fiber recovery, their renewable origin, availability as agricultural residues, and ability to enhance composite properties make them promising candidates for sustainable applications, particularly in lightweight automotive components [6,18].

**Figure 3:**
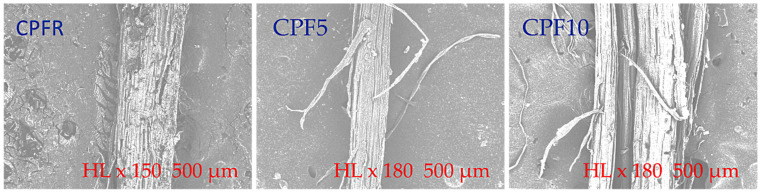
SEM micrographs of the longitudinal surface of *Carica papaya* fibers extracted by water retting (CPFR), 5 wt.% NaOH treatment (CPF5), and 10 wt.% NaOH treatment (CPF10).

SEM observations (Figure 3) revealed that CPFR fibers exhibited a relatively rough surface covered with pectic residues and natural impurities. After alkaline treatment, the fiber surfaces became cleaner and more homogeneous, indicating the effective removal of amorphous constituents. CPF5 fibers displayed moderate surface roughness favorable for mechanical interlocking with the polyester matrix, whereas CPF10 fibers showed the onset of fibrillation resulting from the more severe alkaline attack. This fibrillation increases the available surface area for matrix adhesion; however, excessive treatment may weaken fiber bundles and reduce structural integrity.

The alkaline treatment also modified the geometrical characteristics of the fibers. The average fiber diameter decreased from 260 ± 60 µm for CPFR to 180 ± 30 µm for CPF5, before slightly increasing to 220 ± 50 µm for CPF10. This reduction was mainly associated with the removal of external layers rich in amorphous compounds. The slight diameter increase observed for CPF10 may be related to fibrillation and partial rearrangement of elementary fibers after severe chemical exposure. The fibers retained lengths compatible with the carding process, with average lengths ranging from 67 to 73 mm after nonwoven mat preparation.

**Figure 4:**
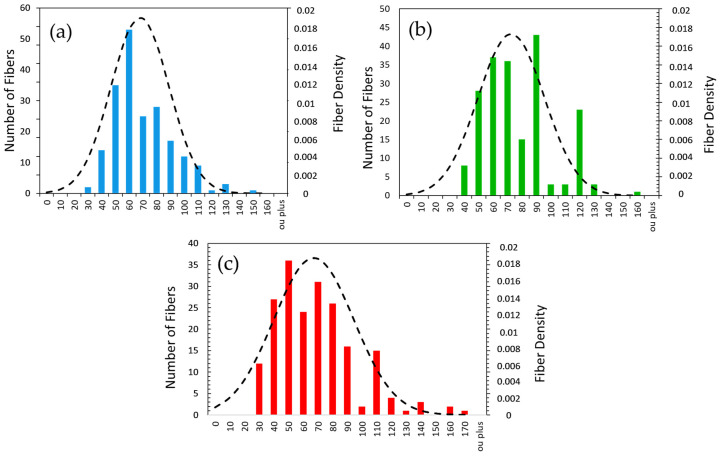
Fiber length distributions of *Carica papaya* fibers contained in the nonwoven reinforcements: (**a**) CPFR, (**b**) CPF5, and (**c**) CPF10.

Fiber length (Figure 4) is a key parameter governing nonwoven cohesion, fiber-matrix load transfer, and energy absorption [26,27,28,29]. The analysis of approximately 200 fibers for each extraction condition showed that the 5 wt.% NaOH treatment (CPF5) produced the highest average fiber length (72.6 ± 23 mm), with a distribution peak around 90 mm. This result reflects improved fiber bundle individualization without excessive degradation. In contrast, the 10 wt.% NaOH treatment (CPF10) yielded an average length of 67.2 ± 27 mm, comparable to that of water-retted fibers (67.8 ± 21 mm), but with a broader distribution ranging from 5 to 170 mm, indicating partial fiber degradation under severe alkaline conditions. Consequently, CPF5 fibers, which combined the greatest average length with good cardability, promoted better fiber entanglement and more efficient load transfer, consistent with their superior mechanical and impact performances [25,30]. Compared with conventional natural fibers such as hemp, jute, and sisal, CP fibers exhibited relatively large lengths, which is advantageous for automotive interior applications.

**Figure 5:**
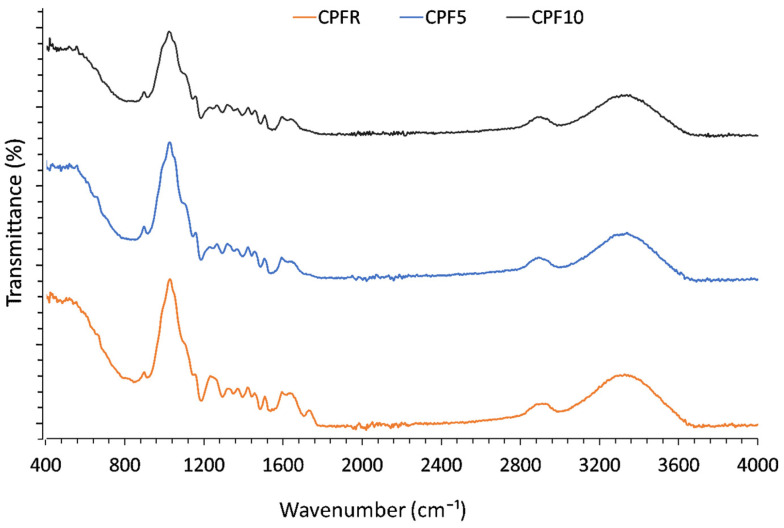
FTIR spectra of CPFR, CPF5, and CPF10 fibers, illustrating the evolution of characteristic absorption bands as a function of alkaline treatment.

FTIR analysis was performed to investigate the chemical modifications induced by the different extraction methods. The spectra of CPFR, CPF5, and CPF10 fibers are presented in Figure 5. The spectra displayed the characteristic absorption bands of lignocellulosic materials. Although NaOH treatment is a well-established chemical modification method for natural fibers, the objective of this analysis was to evaluate the structural changes induced by the extraction process and their influence on fiber–matrix compatibility rather than to claim a new chemical modification route.

A broad band observed at approximately 3320–3340 cm^−1^ was assigned to O-H stretching vibrations associated with cellulose and absorbed water [31,32,33]. Following alkaline treatment, a reduction in hygroscopicity was observed, indicating the partial removal of hydrophilic constituents and improved compatibility with the unsaturated polyester (UP) matrix [27]. The bands located between 2885 and 2910 cm^−1^, attributed to C-H stretching vibrations, showed a decrease in intensity, indicating partial modification of these chemical groups [31].

A major change occurred at 1729 cm^−1^, corresponding to carbonyl (C=O) groups from pectins and hemicelluloses [31,32,33]. The disappearance of this peak after alkaline treatment confirmed the removal of these hydrophilic constituents, which are known to reduce fiber-matrix adhesion [19,34]. The absorption bands at 1593, 1506, and 1420 cm^−1^, associated with lignin structures, exhibited reduced intensities after treatment, indicating partial delignification [7,35,36,37]. These changes confirm that NaOH treatment modifies the fiber surface chemistry by removing non-cellulosic components, thereby enhancing potential interactions with the UP matrix.

This modification exposed cellulose microfibrils and increased the availability of reactive hydroxyl groups, improving potential interfacial interactions with the polyester matrix. Thus, the significance of FTIR analysis in this study lies in establishing the relationship between fiber chemical modification, interfacial behavior, and composite performance.

Additional bands at 1318 cm^−1^ and 1265–1230 cm^−1^ (CH_2_ and acetyl groups), 1155 cm^−1^ (C-O-C), 1026 cm^−1^ (C–O), and 896 cm^−1^ (β-glycosidic linkages) were assigned to hemicellulose and cellulose structures [27,28,29,33]. Overall, the FTIR results confirm the effectiveness of the extraction treatments in tailoring CP fiber surface chemistry for UP composite manufacturing, while the novelty of this work relies on the integration of modified fibers, carded nonwoven architecture, and polymer processing rather than on the NaOH treatment itself.

To quantify hemicelluloses removal, the absorbance ratio A_1730_/A_1026_ decreased from 0.81 for CPFR to 0.37 for CPF10, confirming significant hemicelluloses removal and partial delignification [13,27,29,30]. This reduction was correlated with improved fiber–matrix compatibility, as the elimination of amorphous constituents increased cellulose exposure and surface roughness, thereby enhancing hydrogen bonding and mechanical interlocking with the polyester resin. Therefore, the 5 wt.% NaOH treatment improved interfacial adhesion through three complementary mechanisms: (i) reduction in hydrophilic constituents, (ii) exposure of cellulose microfibrils, and (iii) increased surface roughness. Compared with other plant fibers reported in the literature [28,33], CP fibers exhibited favorable surface modifications for composite reinforcement applications.

The physical properties of the fibers (Table 2) were also strongly affected by alkaline treatment. Fiber density increased from 0.72 g cm^−3^ for CPFR to approximately 1.07 g cm^−3^ for CPF10, indicating a more compact cellulose-rich structure. Simultaneously, water absorption decreased dramatically from 225% for CPFR to 75% for CPF5 and 47% for CPF10. This reduction in hydrophilicity is particularly beneficial for polyester-based composites because it limits fiber swelling and improves dimensional stability at the fiber–matrix interface.

The tensile properties of individual fibers (Table 2) showed that the 5 wt.% NaOH treatment produced the best performance. Tensile strength increased from 116.9 MPa for CPFR to 277.0 MPa for CPF5, while Young’s modulus increased from 2.69 to 4.52 GPa. When the NaOH concentration was increased to 10 wt.%, a slight reduction was observed (218.0 MPa and 4.20 GPa), suggesting localized degradation of the cellulose structure. Consequently, CPF5 represented the best compromise between surface purification and preservation of fiber integrity.

Overall, these results demonstrate that the extraction process strongly influences the morphology, chemical composition, physical characteristics, and mechanical behavior of CP fibers. The optimized 5 wt.% NaOH treatment provided the best balance between removal of non-cellulosic components, preservation of cellulose integrity, improved surface activation, and mechanical reinforcement potential. These modifications are expected to directly affect mat impregnation, fiber–matrix adhesion, and the final performance of the composites developed in this study.

### 3.2. Density, Porosity, and Water Absorption of the Composites

The physical properties of the CP fiber-reinforced polyester composites are presented in Table 3. The results demonstrate that the fiber extraction process significantly influenced composite density, porosity, and water absorption behavior (*p* < 0.05). CPF10UP exhibited the highest hydrostatic and geometric densities, reaching 1.161 and 1.014 g cm^−3^, respectively, compared with 0.966 and 0.812 g cm^−3^ for CPFRUP. This increase reflects improved compaction and more efficient polyester impregnation after alkaline treatment. The removal of non-cellulosic constituents, including hemicelluloses, pectins, waxes, and extractives, likely promoted fiber fibrillation and enhanced fiber-matrix contact, leading to a denser composite structure. Similar trends have been reported for natural fiber composites reinforced with hemp, flax, and *Triumfetta cordifolia* fibers [5,11,28].

The porosity evolution showed a different behavior, with CPF5UP exhibiting the highest value (23.36%), compared with 15.96% for CPFRUP and 12.67% for CPF10UP. The moderate alkaline treatment (5 wt.% NaOH) likely induced partial removal of amorphous components and increased fiber surface roughness, generating additional voids that were not completely filled by the polyester matrix. Similar observations have been reported by Marichelvam et al. [38]. Although high porosity may reduce mechanical performance, it can provide functional advantages for acoustic applications because interconnected pores promote sound dissipation and attenuation [5,22,28,29]. Therefore, CPF5UP may be suitable for automotive components where acoustic comfort is prioritized.

In contrast, CPF10UP exhibited the lowest porosity, indicating a more homogeneous internal structure and improved resin penetration. The higher NaOH concentration promoted more efficient fiber cleaning and reduced interfacial defects, which agrees with SEM observations showing stronger fiber–matrix interactions. Water absorption results confirmed this improvement, with CPF10UP showing the lowest uptake (2.62%), compared with 14.09% for CPFRUP and 17.16% for CPF5UP. This reduction is mainly attributed to the removal of hydrophilic hemicelluloses, reduced open porosity, and enhanced interfacial adhesion, which limit moisture diffusion pathways [13,19,38]. The decrease in accessible hydroxyl groups after alkaline treatment may also contribute to the lower affinity of treated fibers for water.

Overall, the 10 wt.% NaOH treatment provided the most favorable physical properties by combining high density, low porosity, and excellent moisture resistance. These characteristics are particularly advantageous for structural and semi-structural automotive applications exposed to humid environments. However, the comparison between CPF5UP and CPF10UP highlights the importance of balancing morphology and functionality: CPF10UP provides superior environmental resistance, whereas CPF5UP offers higher porosity that may be beneficial for acoustic damping applications. Thus, alkaline treatment represents an effective strategy for tailoring CP fiber/polyester composites according to specific performance requirements.

### 3.3. Shore D Hardness of the Composites

**Figure 6:**
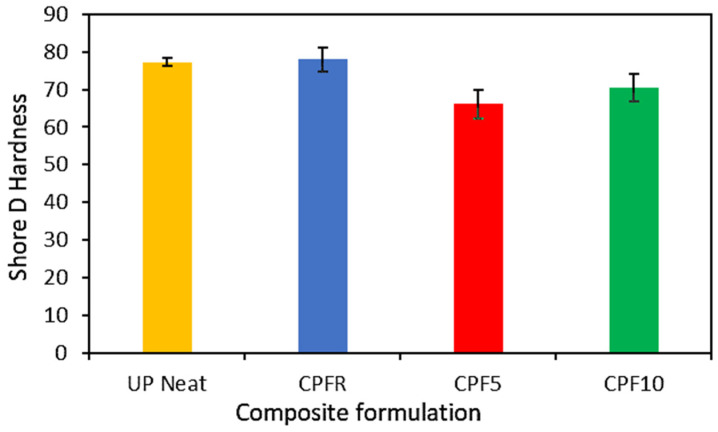
Shore D hardness of neat polyester and CP fiber-reinforced polyester composites.

Figure 6 presents the Shore D hardness values of the developed composites and neat polyester resin, while the corresponding numerical results are reported in Table 3. The incorporation of CP fibers affected composite hardness depending on the extraction process. CPFRUP exhibited a hardness value of 77.93 Shore D, statistically equivalent to that of neat polyester resin (77.21 Shore D), indicating that the addition of water-retted fibers did not significantly alter the local resistance to indentation.

In contrast, alkaline treatment led to a noticeable reduction in hardness. CPF5UP exhibited the lowest value (66.07 Shore D), corresponding to a decrease of approximately 15% compared with neat polyester resin, whereas CPF10UP retained an intermediate value (70.50 Shore D). This reduction is attributed to the microstructural modifications induced by alkaline treatment. The removal of hemicelluloses, pectins, and part of the lignin promoted fiber fibrillation and increased the specific surface area. Although these phenomena generally improve fiber-matrix adhesion and global mechanical properties, they may also generate microvoids and locally less compact regions, thereby reducing indentation resistance [24,35]. Furthermore, the increase in fiber surface roughness and structural heterogeneity after NaOH treatment can decrease the local packing efficiency of the composite, which directly affects Shore D hardness.

These observations are consistent with previous studies. Sawpan et al. [38] reported that alkaline treatment improves interfacial adhesion in hemp/polyester composites but may reduce hardness when the fiber structure becomes more open. Similarly, Marichelvam et al. [36] associated hardness reduction with increased internal porosity. In agreement with these findings, the higher porosity observed for CPF5UP (23.36%) likely increased the number of localized stress relaxation zones, reducing resistance to indentation.

Despite its higher density and lower water absorption, CPF10UP remained less hard than CPFRUP, suggesting that severe alkaline treatment may also alter the local rigidity of the fibrous network. However, the higher hardness of CPF10UP compared with CPF5UP indicates that the 10 wt.% NaOH treatment improved resin impregnation and reduced internal defects, partially compensating for the softening effect caused by fiber modification. Nevertheless, all hardness values remained within the range typically reported for lignocellulosic fiber-reinforced polyester composites (60–85 Shore D) [35].

Therefore, although alkaline treatment slightly reduced Shore D hardness, CPF5UP and CPF10UP maintained hardness levels suitable for semi-structural applications. This behavior highlights the trade-off between surface hardness and other functional properties, as the reduction in indentation resistance was accompanied by improved fiber-matrix interactions, reduced moisture sensitivity, and enhanced energy dissipation capability. More importantly, this reduction was accompanied by substantial improvements in overall mechanical performance and impact resistance, highlighting a favorable balance between local stiffness and energy dissipation capability.

### 3.4. Tensile and Flexural Properties

**Figure 7:**
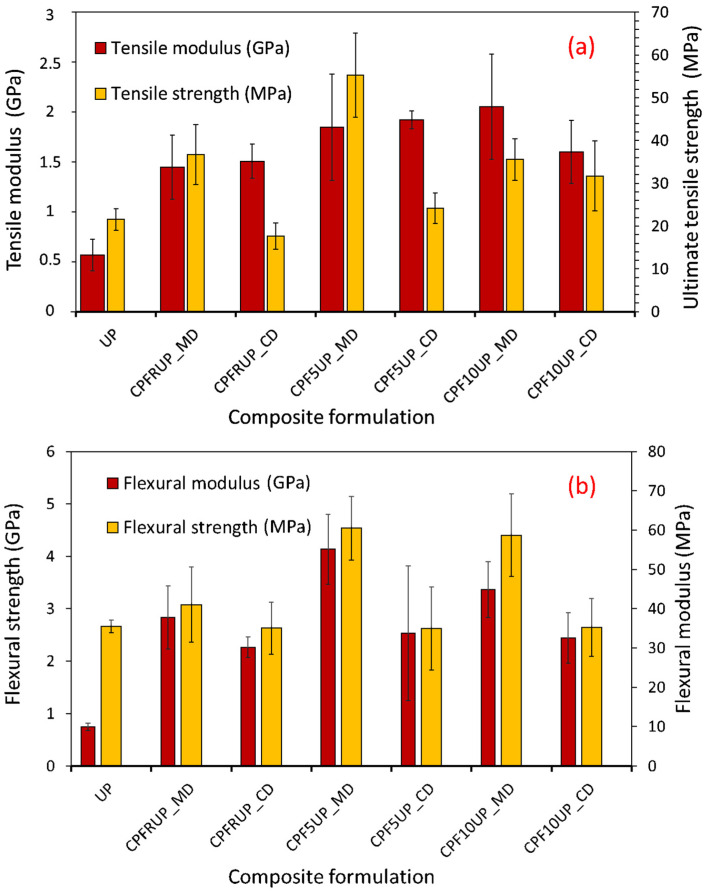
Mechanical properties of *Carica papaya* fiber-reinforced polyester composites: (**a**) tensile modulus and tensile strength, and (**b**) flexural modulus and flexural strength of nonwoven composites prepared from fibers extracted by different methods and evaluated in the machine (MD) and cross (CD) directions.

The tensile and flexural properties of the CP fiber-reinforced composites are summarized in Table 3 and graphically illustrated in Figure 7. Analysis of variance (ANOVA) revealed that both composite formulation and fiber orientation significantly affected all investigated tensile and flexural properties (*p* < 0.05). For the tensile tests, significant differences were observed for Young’s modulus (F = 12.33; *p* = 2.04 × 10^−7^), tensile strength (F = 25.20; *p* = 2.42 × 10^−11^), and elongation at break (F = 35.48; *p* = 1.80 × 10^−13^). Similarly, the flexural properties were significantly influenced by composite formulation, with F-values of 15.94 (*p* = 9.83 × 10^−9^), 11.13 (*p* = 6.36 × 10^−7^), and 41.51 (*p* = 1.72 × 10^−14^) for flexural modulus, flexural strength, and flexural strain at break, respectively. Multiple comparisons performed using Tukey’s HSD test (*p* < 0.05) are indicated by the statistical grouping letters reported in Table 3. The incorporation of CP fibers substantially enhanced the mechanical performance of the polyester matrix, although the magnitude of this improvement strongly depended on both the alkaline treatment and the preferential orientation of the fibers within the nonwoven reinforcement.

Under tensile loading, specimens tested along the machine direction (MD) systematically exhibited superior mechanical properties compared with those tested along the cross direction (CD), confirming the preferential fiber alignment induced by the laboratory-scale carding process (Figure 7a). This anisotropic behavior demonstrates that the carding operation successfully oriented a substantial proportion of the fibers along the MD, thereby promoting more efficient load transfer between the reinforcement and the polyester matrix. For all formulations, MD specimens exhibited significantly higher tensile strengths than CD specimens, reaching 55.29 MPa versus 24.20 MPa for CPF5UP and 35.55 MPa versus 31.74 MPa for CPF10UP. Similar effects of fiber orientation on the mechanical performance of automotive nonwoven composites were reported by Elseify et al. [39], who demonstrated that the alignment and nature of leaf fibers significantly influenced the tensile behavior of date palm fiber-reinforced composites. Such anisotropic behavior is characteristic of composites reinforced with preferentially oriented natural fibers and has been widely reported for flax-, hemp-, kenaf-, and *Triumfetta cordifolia*-based composites [5,7,22,37,40,41].

Among all formulations, CPF5UP_MD exhibited the highest tensile strength, reaching 55.29 ± 9.80 MPa, corresponding to an increase of approximately 156% compared with neat polyester (21.56 MPa, Table 4). This remarkable improvement is directly related to the superior intrinsic properties of CPF5 fibers, whose individual tensile strength was previously identified as the highest among the investigated fiber types. The 5 wt.% NaOH treatment therefore appears to provide the optimum balance between the removal of amorphous constituents and the preservation of the cellulose microfibrillar structure. As summarized in Table 4, CPF5UP_MD also exhibited the largest overall improvements in Young’s modulus (+225%), flexural strength (+70.4%), and flexural modulus (+456%) compared with neat polyester, confirming the effectiveness of the moderate alkaline treatment. Similar conclusions have been reported by several authors [36,37,38,39], confirming that moderate alkaline treatment efficiently removes hemicelluloses, pectins, waxes, and other surface impurities while maintaining the mechanical integrity of lignocellulosic fibers. Conversely, increasing the NaOH concentration to 10 wt.% resulted in a lower tensile strength (35.55 MPa for CPF10UP_MD), despite the highest Young’s modulus (2.05 GPa). This behavior suggests that the more severe alkaline treatment induced localized degradation of the cellulose microfibrils, thereby reducing the load-bearing capacity of the fibers while simultaneously increasing composite stiffness through stronger interfacial bonding. Similar observations have been reported for alkali-treated natural fibers [37,38].

The increase in stiffness and strength was accompanied by a reduction in ductility. Whereas neat polyester exhibited an elongation at break of 6.82%, the reinforced composites displayed values ranging from 1.38% to 4.13%. Among the investigated formulations, CPF5UP_MD maintained the best compromise between strength and ductility, exhibiting an elongation at break of 4.13%, significantly higher than CPF10UP_MD (3.04%). This higher strain-to-failure indicates that progressive fiber pull-out and local stress redistribution mechanisms remained active before final failure. In contrast, CPF10UP_MD exhibited a stiffer and more brittle behavior, most likely resulting from stronger fiber-matrix interactions that restricted energy-dissipation mechanisms. Similar trends have been reported for flax/epoxy composites [40].

A similar influence of alkaline treatment and fiber orientation was observed under flexural loading. The incorporation of CP fibers significantly improved the flexural properties of the polyester matrix, with CPF5UP_MD exhibiting the highest flexural strength (60.52 ± 7.26 MPa), corresponding to an increase of approximately 70% compared with neat polyester (35.51 MPa). CPF10UP_MD showed a comparable value (58.70 ± 11.97 MPa), whereas CPFRUP_MD reached only 41.01 MPa. Likewise, the flexural modulus increased from 2.83 GPa for CPFRUP_MD to 3.36 GPa for CPF10UP_MD and 4.14 GPa for CPF5UP_MD (Figure 7b) These results clearly demonstrate that the 5 wt.% NaOH treatment provides the most favorable balance between flexural stiffness and strength. The superior performance of CPF5UP_MD can be attributed to improved fiber-matrix adhesion, more efficient stress transfer under bending loads, and preservation of the intrinsic mechanical integrity of the fibers. The removal of hemicelluloses, pectins, and waxes improved both the intrinsic stiffness of the fibers and the quality of the fiber-matrix interface [19,36,38]. These findings are consistent with those reported by Elseify et al. [39] for date palm fiber nonwoven composites and by Mewoli et al. [5] for PLA/*Triumfetta cordifolia* composites. Although the flexural properties obtained in the present study are slightly higher, they remain within the range generally reported for well-impregnated natural-fiber nonwoven composites [7,22].

The anisotropic effect of fiber orientation became even more pronounced during flexural loading. Composites tested along the machine direction consistently exhibited substantially higher flexural properties than those tested along the cross direction. For example, the flexural strength of CPF5UP_MD (60.52 MPa) was nearly twice that of CPF5UP_CD (34.93 MPa), illustrating the effectiveness of preferential fiber alignment in promoting efficient stress transfer along the loading direction [5,7,22,29]. In contrast to the tensile behavior, the flexural strain at break followed a different trend, with CPF10UP_MD exhibiting the highest value (2.79%), compared with 2.53% for CPF5UP_MD. This result suggests that the improved structural homogeneity, stronger fiber-matrix interface, and lower porosity of CPF10UP delayed crack initiation and propagation during bending despite the higher stiffness of the composite. Similar behavior was reported by Orhan et al. [24] for borax-treated polyester composites, where more severe surface treatments resulted in higher flexural strain at failure.

Overall, the mechanical results demonstrate two complementary optimization strategies depending on the targeted application. The 5 wt.% NaOH treatment (CPF5UP_MD) provided the most balanced mechanical performance by combining the highest tensile strength (55.3 MPa; 156% relative to neat polyester), the highest elongation at break among the reinforced composites (4.13%), the highest flexural strength (60.5 MPa; 70% relative to neat polyester), and the highest flexural modulus (4.14 GPa). In contrast, the 10 wt.% NaOH treatment (CPF10UP_MD) maximized tensile stiffness (Young’s modulus of 2.05 GPa) and flexural strain at failure (2.79%), reflecting a stiffer and more compact fiber-matrix interface despite a moderate reduction in tensile strength. These findings confirm the strong potential of *Carica papaya* fibers, particularly after moderate alkaline treatment, as sustainable bio-based reinforcements for polyester composites intended for lightweight semi-structural automotive interior applications [41,42,43].

### 3.5. Morphology of Composite Tensile Fracture Surfaces

**Figure 8:**
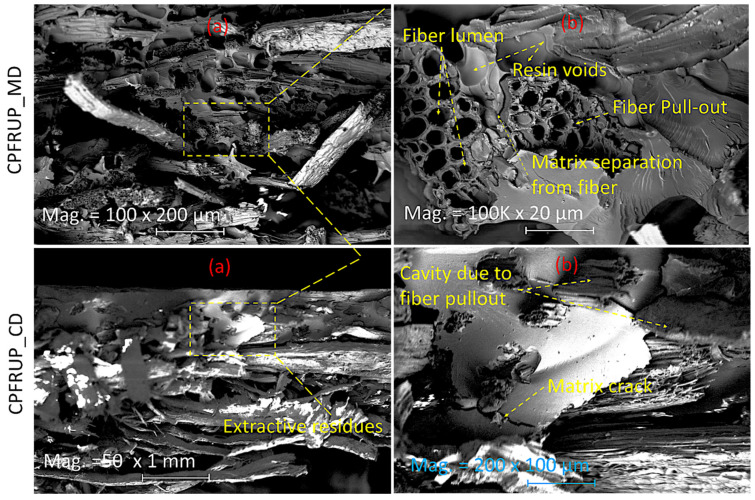
Tensile fracture surface of the CPFRUP composite (water retting): (**a**) general view; (**b**) detail of cavities and fiber pull-out.

**Figure 9:**
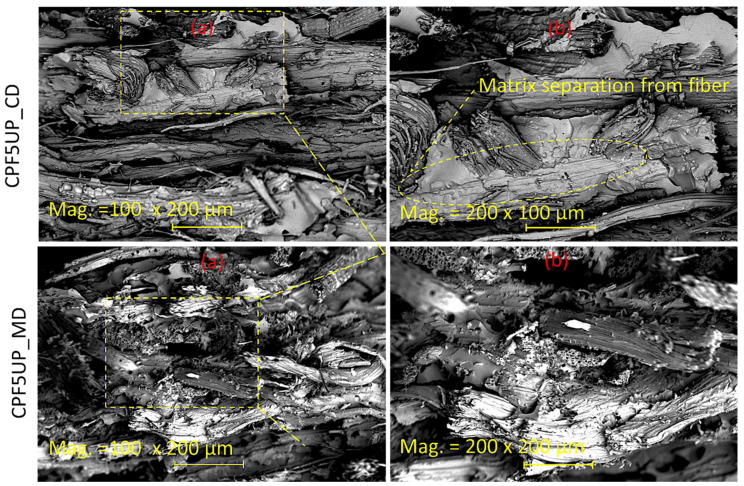
SEM micrographs of the tensile fracture surface of the CPF5UP composite reinforced with 5 wt.% NaOH-treated *Carica papaya* fibers: (**a**) overall view; (**b**) improved fiber-matrix interface.

**Figure 10:**
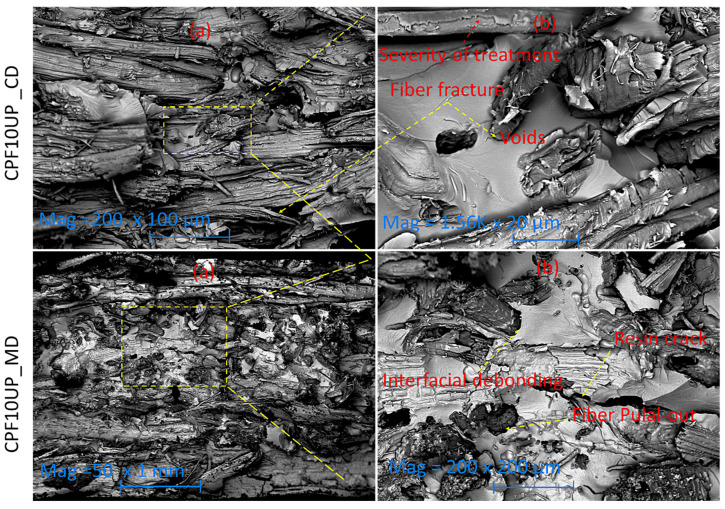
SEM micrographs of the tensile fracture surface of the CPF10UP composite reinforced with 10 wt.% NaOH-treated CP fibers: (**a**) compact fiber-matrix interface; (**b**) fiber fibrillation and brittle fiber fracture.

SEM micrographs of the composite fracture surfaces after tensile testing are presented in Figure 8, Figure 9 and Figure 10. These observations provide valuable insight into the dominant damage mechanisms and help explain the influence of the fiber extraction process on the mechanical behavior of the composites.

For the CPFRUP composites (Figure 8), the micrographs reveal numerous fiber pull-out zones, pronounced fiber-matrix debonding, and residual cavities left after fiber extraction. The fiber surfaces appear partially covered with amorphous residuals, including pectins and hemicelluloses, which likely limited complete wetting of the reinforcement by the polyester matrix. According to previous studies [13,19], water-retted CP fibers retain surface deposits of pectins, hemicelluloses, and other amorphous constituents that reduce matrix wetting and promote crack initiation at the fiber-matrix interface. This weak interfacial adhesion explains the relatively modest tensile and flexural performances observed for CPFRUP composites.

Following the partial removal of non-cellulosic constituents through alkaline extraction with 5 wt.% NaOH, the fracture surfaces of CPF5UP composites exhibited a markedly different morphology (Figure 9). As reported by Olembe et al. [13], alkaline treatment at 5 wt.% NaOH effectively removes non-cellulosic components, including pectins, hemicelluloses, and waxes, while preserving the structural integrity of CP fibers. The fibers appeared more extensively coated with polyester resin, and the number of interfacial voids was significantly reduced. Several regions exhibited simultaneous fracture of both the fibers and the matrix, indicating more efficient stress transfer between the two phases. Mono et al. [19] similarly reported that fibers treated with 5 wt.% NaOH provide the best balance between mechanical strength and flexibility, owing to their cleaner surface and enhanced capacity for mechanical interlocking. The reduced occurrence of fiber UPll-out observed in these micrographs further confirms the improvement in fiber-matrix adhesion resulting from the alkaline treatment. This enhanced interfacial bonding directly explains the superior tensile and flexural performances obtained for the CPF5UP composites. In specimens tested along the machine direction (CPF5UP_MD), the preferential fiber alignment induced by the carding process further enhanced these properties by promoting more efficient load transfer along the reinforcement direction.

For the CPF10UP composites, Figure 10 reveals an even more compact fiber-matrix interface, characterized by homogeneous resin impregnation, reduced porosity, and strong fiber anchorage within the polyester matrix. These microstructural features are consistent with the improved dimensional stability and lower water absorption measured experimentally. However, the more severe alkaline treatment appears to have induced localized damage to the lignocellulosic CP fibers, as evidenced by the presence of brittle fiber fractures and pronounced fibrillation. These observations help explain why CPF10UP_MD exhibited the highest Young’s modulus while displaying a slightly lower tensile strength than CPF5UP_MD. Although the stronger alkaline treatment enhanced interfacial bonding and composite compactness, it also partially reduced the intrinsic load-bearing capacity of the fibers.

The influence of fiber orientation is also clearly visible on the fracture surfaces. Regardless of the composite formulation, the micrographs confirm the strong anisotropy induced by the carding process. Specimens tested along the machine direction (MD) generally exhibited a higher proportion of fractured fibers, reflecting their preferential alignment with the loading direction, indicating efficient stress transfer from the matrix to the reinforcement. In contrast, specimens tested along the cross direction (CD) showed a greater contribution of matrix deformation, interfacial debonding, and fiber UPll-out. Similar observations have been reported by Mewoli et al. [5], who demonstrated that this anisotropic behavior originates from the preferential alignment of fibers along the machine direction during carding. These observations further confirm the significant influence of fiber orientation on the mechanical performance of nonwoven composites.

Overall, the damage mechanisms identified in this study are consistent with those reported for various natural fiber-reinforced composites [37,38,40,44]. Olembe et al. [13] emphasized that the improvement in fiber-matrix adhesion after alkaline treatment primarily results from the removal of amorphous constituents and the increased exposure of cellulose hydroxyl groups, which promote stronger interfacial interactions with the polyester matrix. According to these authors, the mechanical performance of lignocellulosic composites mainly depends on the quality of the fiber-matrix interface, the degree of resin impregnation, and the efficiency of stress transfer from the matrix to the fibers. Mono et al. [19] similarly reported that fibers treated with 5 wt.% NaOH provide the best compromise between surface purification and preservation of fiber mechanical integrity. The present observations confirm that alkaline treatment significantly improves these key parameters in CP fiber-reinforced polyester composites, thereby explaining the superior mechanical performance achieved by the treated-fiber formulations.

Overall, the SEM observations are fully consistent with the physical and mechanical results discussed in the previous sections. The 5 wt.% NaOH treatment provided the most favorable balance between enhanced fiber-matrix interfacial adhesion and preservation of fiber mechanical integrity. In contrast, the 10 wt.% NaOH treatment produced a more compact and stiffer interface but also induced slight degradation of the fibers. These findings are consistent with recent studies [13,14,19,43], further confirming the potential of *Carica papaya* fibers as sustainable reinforcements for high-performance biocomposite applications.

### 3.6. Low-Velocity Impact Resistance

**Figure 11:**
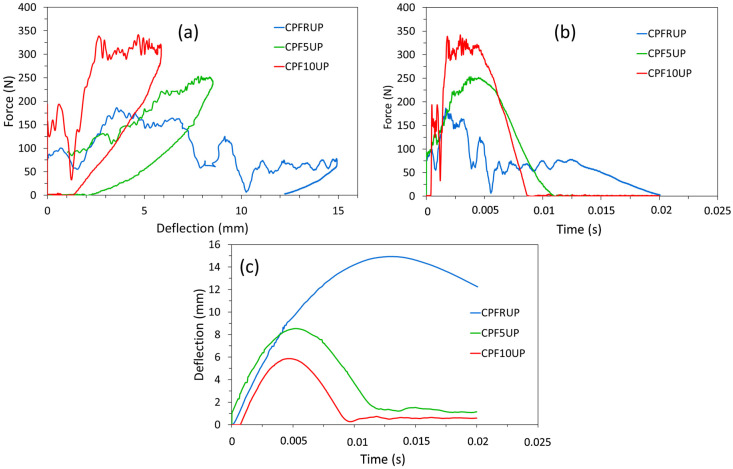
Evolution of the low-velocity impact parameters of CPFRUP, CPF5UP, and CPF10UP composites: (**a**) force-deflection curves; (**b**) force-impact time curves; and (**c**) absorbed energy-impact time curves.

All numerical values are summarized in Table 5. Figure 11a presents the force-deflection curves of the three composites. Three characteristic stages can be identified: an initial peak corresponding to crack initiation and the onset of fiber-matrix debonding, a fluctuating region associated with unstable crack propagation accompanied by fiber UPll-out and interfacial friction, and, in some cases, a second peak related to interlayer delamination [25,39]. These successive stages reflect the progressive activation of the different energy dissipation mechanisms occurring within the nonwoven composite structure under impact loading. Figure 11b shows the corresponding force-time curves. CPF10UP exhibited the shortest contact duration, reflecting its higher dynamic stiffness. In contrast, CPFRUP displayed a longer contact time and more pronounced oscillations, indicating lower internal cohesion and greater energy dissipation through progressive damage mechanisms. The absorbed energy-time curves are presented in Figure 11c. For all formulations, the absorbed energy increased progressively with time until reaching a plateau corresponding to the total dissipated energy. CPF10UP exhibited the steepest slope, indicating a higher dynamic stiffness and a more rapid transfer of impact energy. Conversely, CPFRUP showed a more gradual increase in absorbed energy, reflecting greater deformation and progressive damage development before reaching the maximum absorbed energy.

Indeed, CPFRUP exhibited a relatively low slope and pronounced oscillations after the peak load, indicating limited internal cohesion and progressive damage development. In contrast, CPF5UP displayed a steeper slope and fewer fluctuations, reflecting improved fiber-matrix adhesion and more efficient load transfer during impact. CPF10UP was characterized by a very steep slope, a single high peak load (344 N), and a sharp force drop thereafter, indicating a stiff interface and a more abrupt failure mode. This behavior suggests that excessive interface stiffness may limit damage tolerance by reducing progressive energy dissipation mechanisms. None of the specimens were perforated under the selected impact conditions, unlike the flax/tannin nonwoven composites investigated by Zhu et al. [26], which were perforated at an impact energy of 6.9 J, and the date palm fiber/PP composites studied by Elseify et al. [39]. The absence of perforation confirms the ability of CP fiber composites to delay catastrophic failure under low-velocity impact loading. This behavior demonstrates the superior resistance of the present composites to crack propagation and perforation under low-velocity impact loading. The absence of perforation can also be attributed to the intermediate fiber lengths obtained after carding (67–73 mm), which limit the formation of preferential crack propagation paths. Consequently, the impact energy is dissipated through a combination of matrix cracking, interfacial friction, fiber pull-out, and progressive fiber fracture rather than through complete perforation.

Analysis of the energy-deflection curves (Figure 11c) showed that CPF5UP absorbed the highest amount of energy (2.45 J) over a wider deformation range, whereas CPF10UP reached a comparable energy level more rapidly but at a much lower deflection (2.37 J at 5.87 mm). CPFRUP dissipated an intermediate amount of energy (2.20 J) over a substantially larger deflection (14.94 mm), confirming its more ductile but less resistant behavior. The higher energy absorption of CPF5UP can be attributed to the optimum balance between fiber integrity, surface modification, and controlled interfacial damage, which allows progressive dissipation without premature failure.

Furthermore, the maximum impact force increased from 184 N for CPFRUP to 344 N for CPF10UP, corresponding to an increase of approximately 87%, while the maximum deflection decreased by 61%, from 14.94 mm to 5.87 mm. The residual deflection decreased dramatically from 82.5% of the maximum deflection for CPFRUP (12.3 mm) to only 13% for CPF5UP (1.1 mm) and 10% for CPF10UP (0.6 mm). These results indicate that alkaline-treated composites transitioned from a predominantly plastic response to a more elastic impact behavior [25], in agreement with the findings of Shah et al. [45] for 3D thermoplastic composites. This transition is mainly associated with improved stress transfer efficiency and reduced interfacial defects after alkaline treatment.

CPF5UP exhibited the highest absorbed energy (2.45 J), corresponding to approximately 80% of the incident impact energy, owing to efficient energy dissipation through controlled cracking, fiber pull-out, and interfacial friction. In contrast, CPF10UP absorbed slightly less energy (2.37 J) because a larger fraction of the impact energy was elastically recovered. Consequently, CPF10UP exhibited a much higher impact stiffness (58.6 N mm^−1^) than CPF5UP (29.8 N mm^−1^) and CPFRUP (12.3 N mm^−1^). These trends are consistent with the observations reported by Manaii et al. [25] for flax/Elium^®^ composites. Alkaline treatment improved impact performance by enhancing fiber-matrix adhesion and reducing porosity, thereby limiting crack initiation and increasing interfacial stiffness [25,39]. Similar observations were reported by Hoffmann et al. [42] for natural fiber nonwoven composites, where improved interfacial bonding contributed to greater impact resistance and more efficient energy dissipation.

Overall, CPFRUP absorbed impact energy primarily through plastic deformation and fiber pull-out, as evidenced by the large deflections observed during testing. CPF5UP provided the most favorable balance between stiffness and energy absorption, maximizing impact energy dissipation through controlled cracking and interfacial friction mechanisms. In contrast, the stiffer CPF10UP composite exhibited the highest impact resistance and the best elastic recovery, albeit with a slight reduction in absorbed energy. This difference highlights the influence of treatment severity on impact behavior: moderate alkaline treatment favors damage tolerance, whereas stronger treatment promotes stiffness and structural resistance. This stiffness-absorption trade-off, commonly reported for natural fiber nonwoven composites [26,39,41], highlights the potential of alkali-treated CP fibers for low-velocity impact applications in automotive interiors, including door panels and headliners.

### 3.7. Differential Scanning Calorimetry Analysis (DSC)

**Figure 12:**
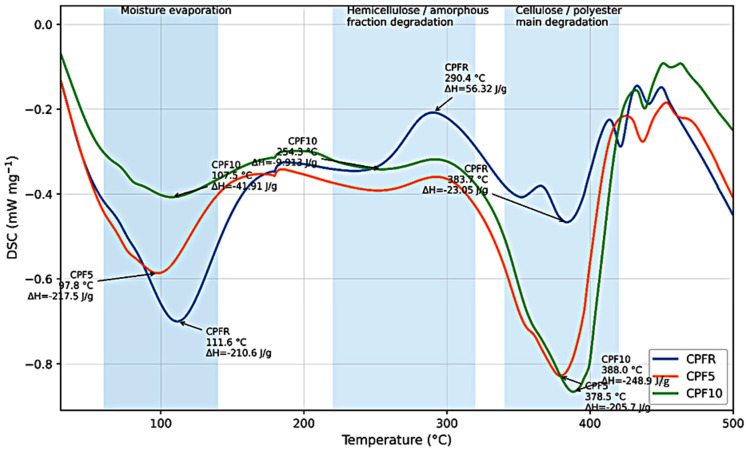
DSC thermograms of polyester composites reinforced with *Carica papaya* fibers (CPFRUP, CPF5UP, and CPF10UP).

The DSC thermograms of the three composites (Figure 12) revealed three characteristic thermal regions: moisture evaporation, degradation of the amorphous fiber constituents, and the main decomposition of the fiber–matrix system. These thermal transitions reflect the progressive degradation of the different composite constituents, including absorbed water, lignocellulosic components, and polyester chains.

A first endothermic peak was observed between approximately 80 and 130 °C and is attributed to moisture evaporation. CPFRUP exhibited a peak at 111.6 °C with an enthalpy of −210.6 J g^−1^, whereas CPF10UP showed a much lower enthalpy value (−41.9 J g^−1^). This substantial reduction indicates a markedly lower water retention capacity after alkaline treatment, which is fully consistent with the water absorption results previously reported (2.62% for CPF10UP compared with 14.09% for CPFRUP). This behavior results from the removal of hydrophilic components, particularly hemicelluloses and pectins, which reduces the number of accessible hydroxyl groups responsible for moisture adsorption. Similar observations were reported by Kabir et al. [46], who demonstrated that the endothermic peak associated with moisture evaporation is more pronounced in untreated natural fibers (typically around 80 °C) and decreases in intensity after alkaline treatment due to the partial removal of hydroxyl-containing constituents responsible for water uptake.

A second thermal event was observed between approximately 220 and 320 °C and is associated with the degradation of hemicelluloses, pectins, and other amorphous constituents. This peak was particularly pronounced for CPFRUP (290.4 °C, 56.3 J g^−1^) but became almost negligible for CPF10UP (254.3 °C, 9.9 J g^−1^). These results indicate that the alkaline treatment effectively removed a large proportion of the thermally unstable amorphous fractions. The disappearance of this thermal transition confirms the selective extraction of less stable components, resulting in a higher relative cellulose content within the treated fibers. Similar findings were reported by Kabir et al. [46] who observed that the characteristic thermal peak associated with hemicellulose degradation (typically occurring between 250 and 290 °C in untreated hemp fibers) disappeared after alkaline treatment or acetylation, confirming the effectiveness of these treatments in removing thermally labile amorphous constituents. Similarly, Su and Wu [47] reported that the incorporation of natural fibers into a polyester matrix modifies the DSC response, with a reduction in thermal events associated with amorphous constituents following fiber surface treatment and improved fiber-matrix compatibility.

The main thermal event, corresponding to the simultaneous degradation of cellulose from the fibers and the polyester matrix, occurred between approximately 350 and 420 °C. CPF10UP exhibited the highest degradation temperature (388.0 °C), compared with 383.7 °C for CPFRUP, indicating improved thermal stability following alkaline treatment. The associated enthalpy also increased markedly with treatment severity, from −23.1 J g^−1^ for CPFRUP to −205.7 J g^−1^ for CPF5UP and −248.9 J g^−1^ for CPF10UP. The higher enthalpy values observed after treatment suggest that a greater amount of thermally stable cellulose-rich structures participated in the degradation process, while improved fiber–matrix adhesion contributed to a more homogeneous heat transfer within the composite. Similar degradation ranges (300–400 °C) and enthalpy variations have been reported by Akter et al. [48] and Su et Wu [47] confirming the beneficial effect of alkaline treatment on both thermal stability and fiber-matrix interfacial bonding.

Overall, the DSC analysis confirms that alkaline treatment improves the thermal behavior of CP fiber/polyester composites through the removal of hydrophilic and thermally unstable amorphous constituents. CPF10UP exhibited the highest thermal stability, as evidenced by its higher degradation temperature and reduced moisture-related thermal transition, whereas CPF5UP provided a particularly attractive balance between thermal stability, mechanical performance, and impact energy absorption. These results highlight the importance of optimizing alkaline treatment severity, since excessive chemical modification improves thermal resistance but may affect fiber integrity and mechanical reinforcement capability. These findings are in good agreement with previous studies on polyester composites reinforced with natural fibers.

### 3.8. Thermogravimetric Analysis (TGA) of the Composites

**Figure 13:**
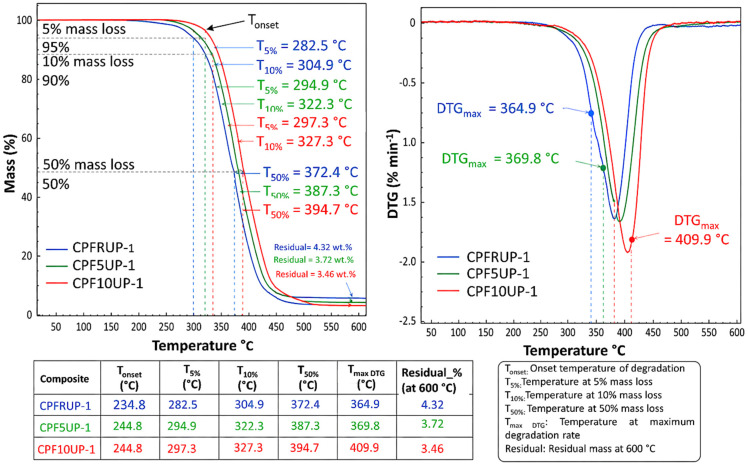
TG and DTG thermograms of polyester composites reinforced with *Carica papaya* fibers: CPFRUP (water retting), CPF5UP (5 wt.% NaOH treatment), and CPF10UP (10 wt.% NaOH treatment).

The TG and DTG curves of the three composites (Figure 13) exhibit the typical thermal degradation behavior associated with the simultaneous decomposition of lignocellulosic components from the fibers and the polyester matrix [13,30,49]. The results clearly indicate that alkaline treatment improved the thermal stability of the composites. The temperature corresponding to 5% weight loss (T_5_%) increased from 282.5 °C for CPFRUP to 297.3 °C for CPF10UP. Similarly, T_10_% increased from 304.9 °C to 327.3 °C, while T_50_% rose from 372.4 °C to 394.7 °C. These results demonstrate that alkaline treatment delayed the onset of thermal degradation by modifying the fiber chemical composition and reducing the proportion of thermally sensitive constituents.

An even more pronounced effect was observed for the maximum degradation temperature (T_max_ DTG), which increased from 364.9 °C for CPFRUP to 369.8 °C for CPF5UP and reached 409.9 °C for CPF10UP, corresponding to a shift of nearly 45 °C. This improvement can be attributed to the partial removal of thermally unstable amorphous components, such as hemicelluloses, pectins, and extractives, during alkaline treatment. Consequently, the treated fibers exhibited a higher relative cellulose content, which contributes to improved thermal resistance. Furthermore, enhanced fiber–matrix interactions may have contributed to limiting heat transfer and delaying the degradation process [49,50].

The char residue remaining after pyrolysis decreased slightly from 4.32% for CPFRUP to 3.46% for CPF10UP. This reduction may be associated with the removal of lignin-containing fractions and extractable compounds during alkaline treatment, leading to a cleaner and more homogeneous fibrous reinforcement [50].

TGA results confirmed that alkaline treatment positively influenced the thermal behavior of the composites, with CPF10UP exhibiting the highest thermal resistance, as indicated by its superior T_max_ DTG value. These results are consistent with the DSC observations. However, a balance between thermal and mechanical performance was observed. Although CPF10UP showed the highest thermal stability, CPF5UP exhibited the best overall mechanical performance under tensile and flexural loading. This behavior suggests that increasing NaOH concentration enhances the removal of non-cellulosic constituents and thermal resistance but may also induce partial modification of the fiber structure, affecting mechanical reinforcement efficiency. Therefore, the 5 wt.% NaOH treatment provided the most favorable compromise between thermal stability, preservation of fiber integrity, and mechanical performance.

### 3.9. Comparative Performance and Benchmarking of Natural Fiber Nonwoven Composites for Automotive Applications

The combined analysis of the physical, mechanical, impact, and thermal properties demonstrates that alkaline treatment significantly improves fiber-matrix adhesion, composite compactness, and thermal stability. CPF10UP exhibited the most compact structure, with the lowest porosity (12.7%) and water absorption (2.62%). In contrast, CPF5UP achieved the best overall mechanical performance, reaching a tensile strength of 55.3 MPa and a flexural strength of 60.5 MPa. CPF10UP maximized tensile stiffness (2.05 GPa), peak impact force (344 N), and thermal stability, with a T_max_ DTG value of approximately 410 °C. These results confirm that treatment severity governs a balance between structural densification, reinforcement efficiency, and preservation of fiber integrity. These findings are consistent with those reported by Mewoli et al. [5] for PLA/Triumfetta cordifolia composites, where a strong MD/CD anisotropy was observed and porosity levels above 60% were shown to negatively affect mechanical performance.

Table 6 positions the developed composites against industrial and academic benchmarks. CPF5UP (MD) outperformed conventional PP/natural fiber composites, including date palm and sisal fiber composites, as well as commercial FlexForm panels, exhibiting a tensile strength of 55.3 MPa compared with 21–32 MPa and a flexural modulus of 4.14 GPa compared with 2.4–2.9 GPa. CPF10UP distinguished itself by its low porosity (13%) and very low water absorption (2.62%). This superior moisture resistance is attributed to the combined effects of hemicellulose removal, reduced accessible hydroxyl groups, and improved fiber–matrix contact, which limit water diffusion pathways within the composite.

The comparative analysis presented in Table 6 shows that CPF5UP (MD) achieved the best overall mechanical performance, with a tensile strength of 55.3 MPa (156% compared with neat polyester), a flexural strength of 60.5 MPa (70%), and a flexural modulus of 4.14 GPa (460%). These values exceed those reported for industrial references such as FlexForm panels and for composites reinforced with conventional natural fibers, including flax, date palm, and sisal. The superior performance of CPF5UP can be explained by the optimal compromise between fiber purification, surface activation, and preservation of fiber length, which promotes efficient stress transfer without inducing excessive structural degradation. In contrast, CPF10UP exhibited the lowest porosity (13%) and water absorption (2.62%), which are advantageous characteristics for applications exposed to humid environments.

Overall, *Carica papaya* fibers demonstrate strong potential as a sustainable and high-performance reinforcement for automotive interior applications. The combination of excellent mechanical properties, low moisture sensitivity, and enhanced thermal stability highlights their suitability for lightweight semi-structural components used in door panels, headliners, and interior trim systems. Compared with conventional natural fiber reinforcements, CP fibers provide an attractive alternative by combining competitive mechanical performance with the environmental benefits associated with agricultural waste valorization.

Consequently, the 5 wt.% NaOH treatment appears to provide the most favorable balance for automotive interior components subjected to mechanical loading, such as door panels and headliners, whereas the 10 wt.% NaOH treatment is better suited for applications requiring enhanced moisture resistance and superior thermal stability [21,42,43]. This distinction highlights that the optimum treatment depends on the targeted application: CPF5UP favors impact tolerance and structural performance, while CPF10UP provides improved dimensional stability and environmental durability. These findings highlight the strong potential of *Carica papaya* fibers as sustainable reinforcements for bio-based composites intended for automotive interior applications, in line with the principles of the circular economy and sustainable material development [43].

#### Comparison with Existing Natural Fiber-Reinforced Polyester Composites and Automotive Door Panel Materials

The mechanical performance of the developed *Carica papaya*/unsaturated polyester composites was compared with materials commonly employed for automotive interior door panels to evaluate their potential for lightweight semi-structural applications. Table 7 summarizes the typical applications, advantages, limitations, and relative positioning of the developed composites with respect to conventional materials currently used in the automotive industry.

The comparison indicates that the developed CPF5UP and CPF10UP composites exhibit a combination of mechanical performance, lightweight characteristics, and environmental sustainability comparable to commercially used natural fiber composites for automotive interior applications. While glass fiber-reinforced materials still provide superior structural strength, the proposed composites offer lower environmental impact and originate from an abundant agricultural residue. CPF5UP is particularly suitable where tensile and flexural performance are required, whereas CPF10UP is more appropriate for humid service environments owing to its improved moisture resistance, impact behavior, and thermal stability. These results further support the potential of *Carica papaya* fiber-reinforced polyester composites as sustainable alternatives for semi-structural automotive door panel applications.

### 3.10. Performance Summary: Radar Chart Analysis

**Figure 14:**
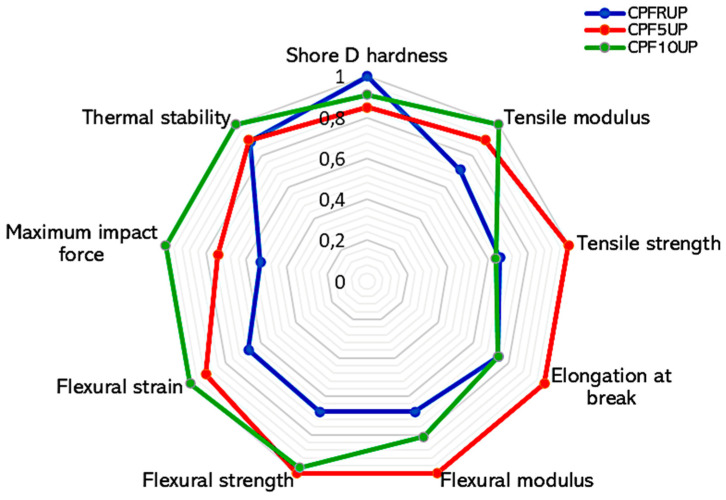
Radar chart summarizing the normalized physical, mechanical, impact, and thermal properties of CPFRUP, CPF5UP, and CPF10UP composites. Values were normalized with respect to the maximum value of each property (score = 1 for the best-performing composite).

To simultaneously visualize all the investigated properties and facilitate comparison among the three composite formulations, a radar chart based on normalized physical, mechanical, thermal, and impact properties was constructed (Figure 14). The values were normalized with respect to the maximum value of each property, such that the best-performing composite received a score of 1. This graphical representation highlights the overall influence of the fiber extraction process on composite performance and provides a comprehensive assessment of the strengths and limitations of each formulation. The radar approach allows the identification of performance trade-offs that cannot be fully captured when individual properties are analyzed separately.

The radar chart provides a comprehensive overview of the relative performance of the three composite formulations and highlights two distinct optimization strategies. CPF5UP exhibited the best overall mechanical performance, particularly in terms of tensile strength, tensile ductility, flexural strength, and flexural modulus, while also maintaining a high capacity for impact energy absorption. This behavior results from the optimal balance between fiber surface modification, preservation of fiber length, and efficient stress transfer within the composite structure. In contrast, CPF10UP excelled in tensile stiffness, flexural strain at failure, peak impact force, and thermal stability. The superior stiffness and thermal resistance of CPF10UP are mainly associated with enhanced fiber purification, reduced porosity, and stronger interfacial cohesion after severe alkaline treatment.

Furthermore, CPF10UP was characterized by the lowest porosity and water absorption values, reflecting its superior resistance to moisture and improved dimensional stability. However, its Shore D hardness remained slightly lower than that of CPFRUP. This observation confirms that the improvement of global composite performance does not necessarily result in increased surface hardness, as hardness is mainly governed by local compactness and surface resistance to indentation. Overall, these results confirm that the 5 wt.% NaOH treatment provides the most favorable balance for applications requiring high mechanical performance, whereas the 10 wt.% NaOH treatment is better suited for applications demanding enhanced moisture resistance, dimensional stability, and thermal durability.

**Figure 15:**
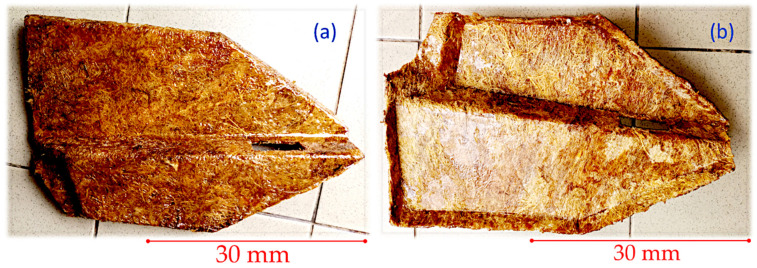
Prototype automotive door panel manufactured from CPF5UP composite (*CP* fibers/polyester): (**a**) inner side; (**b**) outer side.

Considering these complementary results, CPF5UP was identified as the optimal formulation for proof-of-concept implementation. Accordingly, it was selected for the fabrication of a prototype automotive door panel (Figure 15) because it offered the best compromise between mechanical performance, impact energy absorption, and dimensional stability while maintaining the lightweight characteristics required for automotive applications. The selection of CPF5UP demonstrates that moderate alkaline treatment is sufficient to activate the fiber surface while avoiding excessive degradation of the cellulose framework. This demonstrator highlights the application potential of *Carica papaya* fibers for automotive interior components. The attractive specific properties of CPF5UP, including its low density, high flexural stiffness, and good impact resistance, make it a promising candidate for lightweight semi-structural parts such as door panels, headliners, and seat backrests.

Beyond validating the material at the laboratory scale, the successful realization of this demonstrator confirms the technological feasibility of integrating *Carica papaya*-based biocomposites into engineering applications. The prototype represents an important transition from material characterization toward application-oriented validation, demonstrating that agricultural residues can be converted into functional engineering materials. This work therefore demonstrates the feasibility of valorizing tropical agricultural residuals as sustainable engineering materials for automotive applications. By transforming end-of-life *Carica papaya* pseudostems into high-performance biocomposites, this approach contributes to waste reduction, resource efficiency, and the development of environmentally friendly materials, fully aligning with the principles of the circular economy and sustainable development.

### 3.11. Economic Feasibility and Valorization Potential

Beyond the technical performance achieved, the valorization of *Carica papaya* pseudostem fibers presents significant economic potential. The raw material used in this study is an abundant agricultural residue generally left in the field after fruit harvesting, resulting in low procurement costs. According to FAOSTAT, Cameroon produces approximately 43,000 t of papaya annually, generating a substantial amount of pseudostem biomass available for valorization [12]. Since each papaya plant generally undergoes a single fruiting cycle before removal, this biomass represents an abundant and continuously renewable lignocellulosic resource. The extraction yield obtained in this study (10.79–13.10%) indicates that approximately 11–13 kg of dry fibers can be recovered from 100 kg of pseudostem biomass. Although moderate, this yield remains economically relevant because the raw material is an agricultural by-product requiring no additional cultivation inputs. Previous studies have reported that papaya pseudostems contain approximately 35–45 wt.% fibrous material, depending on plant maturity, extraction method, and moisture content, suggesting a considerable theoretical fiber recovery potential [13]. Moreover, the main processing steps, including fiber collection, extraction, drying, and carding, rely on simple technologies suitable for local implementation. Similar valorization strategies have demonstrated the potential of agricultural residues as low-cost reinforcement sources for sustainable composites [3,4].

The incorporation of these fibers into nonwoven composites offers an opportunity to create additional value within the papaya production chain while promoting circular bioeconomy approaches. Considering the current underutilization of papaya pseudostem biomass, even partial recovery of this resource could provide sufficient feedstock for producing nonwoven reinforcement mats for automotive interior components and other semi-structural applications. Compared with synthetic reinforcements, natural fibers present lower density (typically 1.2–1.5 g cm^−3^) and lower environmental impacts, which are advantageous for lightweight automotive applications [6,9]. Unlike glass fibers, whose production involves energy-intensive melting processes, papaya fibers originate from agricultural waste, meaning that the major economic contributions are related to collection, chemical treatment, washing, drying, carding, and composite processing rather than raw-material acquisition.

The results obtained demonstrate that *Carica papaya* fiber-reinforced composites, particularly CPF5UP, achieve performance levels compatible with automotive interior applications. The CPF5UP composite exhibited tensile strength (55.3 MPa), flexural strength (60.5 MPa), and flexural modulus (4.14 GPa), values comparable to other natural fiber/polyester composites, such as hemp/polyester and kenaf/polyester systems reported in the literature [35,38]. These properties are also within the range required for lightweight non-structural automotive components, where natural fiber composites are increasingly considered as alternatives to conventional glass fiber-reinforced materials [6,39]. Assuming a composite formulation containing approximately 30 wt.% fiber reinforcement, 1 metric ton of recovered dry papaya fibers could theoretically produce approximately 3.3 metric tons of composite material, excluding processing losses. This estimation highlights the significant potential for converting a currently underutilized agricultural residue into value-added engineering materials.

The combination of resource availability, low-cost feedstock, and competitive mechanical performance suggests promising techno-economic feasibility. Furthermore, local processing of papaya fibers could generate additional economic benefits by creating employment opportunities associated with biomass collection, fiber extraction, and composite manufacturing, particularly in rural agricultural regions. However, a complete economic assessment should consider key parameters such as fiber yield, chemical consumption during alkaline treatment, energy demand, processing costs, and supply chain logistics. Detailed techno-economic analyses, including life-cycle assessment, cost modeling, industrial-scale production scenarios, transportation requirements, and long-term durability evaluation under service conditions, are still required to fully establish the commercial competitiveness of CP fiber composites.

## 4. Conclusions and Future Perspectives

This study investigated the influence of *Carica papaya* (CP) pseudostem fiber extraction methods on the physical, mechanical, thermal, and impact properties of nonwoven polyester composites intended for automotive interior applications. Three extraction methods were compared: water retting (CPFR) and alkaline treatments with 5 wt.% NaOH (CPF5) and 10 wt.% NaOH (CPF10).

The results demonstrated that alkaline treatment significantly modified fiber morphology, removed hydrophilic amorphous constituents such as hemicelluloses and pectins, and improved fiber–matrix adhesion. CPF10UP exhibited the most compact structure, with the lowest porosity (12.7%) and water absorption (2.62%), highlighting the effectiveness of severe alkaline treatment for applications exposed to humid environments. In contrast, CPF5UP delivered the best overall mechanical performance, achieving a tensile strength of 55.3 MPa (156% compared with neat polyester), a flexural strength of 60.5 MPa (70%), and a flexural modulus of 4.14 GPa (460%). The radar chart clearly revealed two distinct optimization strategies: CPF5UP dominated in tensile and flexural properties, whereas CPF10UP excelled in tensile stiffness (2.05 GPa), peak impact force (344 N), and thermal stability (409.9 °C). These results demonstrate that treatment severity must be optimized according to the targeted application, since improved thermal and environmental resistance may occur at the expense of mechanical reinforcement efficiency.

Low-velocity impact testing showed that the maximum impact force increased from 184 N for CPFRUP to 344 N for CPF10UP, while residual deformation decreased dramatically from 82.5% to only 10%, indicating a transition from a predominantly plastic response to a more elastic behavior following alkaline treatment. DSC and TGA analyses further confirmed that the progressive removal of amorphous constituents enhanced the thermal stability of the composites as the NaOH concentration increased. The combined improvement in impact resistance and thermal behavior confirms the effectiveness of alkaline treatment in modifying the fiber architecture and strengthening the fiber–matrix interface.

Overall, the 5 wt.% NaOH treatment provided the most favorable balance for applications requiring high mechanical performance, such as door panels, headliners, and seat backrests, whereas the 10 wt.% NaOH treatment appears more suitable for applications requiring enhanced moisture resistance, maximum thermal stability, and superior impact stiffness. These findings demonstrate that fibers extracted from *Carica papaya* pseudostems, an abundant agricultural residual in tropical regions, constitute a promising, sustainable, and high-performance bio-based reinforcement for polyester composites. The successful transformation of this agricultural residue into functional engineering materials highlights its potential contribution to lightweight automotive design and circular material strategies.

The promising laboratory-scale results obtained in this study open several perspectives for future development, both from scientific and industrial viewpoints.

From a scientific perspective, future work will focus on optimizing the architecture of the nonwoven reinforcements, including fiber orientation, areal density, and porosity level, in order to further improve the isotropy of mechanical properties and refine the balance between stiffness, energy absorption, and lightweight performance. Fatigue behavior and hygrothermal aging will also need to be investigated to assess the long-term durability of these composites under realistic automotive service conditions. In addition, hybrid composites combining CP fibers with other natural fibers (flax, hemp) or thermoplastic matrices (PP, PLA, and Elium^®^-based systems) will be explored to tailor material properties and diversify bio-based reinforcement solutions. Such approaches could provide additional opportunities to overcome the intrinsic variability of natural fibers and expand their application range toward more demanding structural components.

From a technological and industrial perspective, future developments will focus on scaling up the manufacturing process. Particular attention will be devoted to exploiting pilot-scale dry carding lines available at CETELOR (Épinal, France) and other industrial facilities to produce wider nonwoven webs at semi-industrial production rates. This scale-up phase will enable the evaluation of process reproducibility, optimization of carding and needling parameters (penetration density, line speed, and number of layers), and validation of the feasibility of continuous production of CP fiber-based nonwoven rolls. Furthermore, these pilot facilities will facilitate the manufacture of larger demonstrators, including automotive door panels and headliners, for testing under conditions representative of real automotive applications in collaboration with industrial partners. These steps are essential to bridge the gap between laboratory validation and industrial implementation.

Finally, the development of fully bio-based composites incorporating plant-derived matrices, together with end-of-life recyclability and circularity assessments, will be investigated to further enhance the attractiveness of these materials for lightweight automotive applications and technical furniture products. Life-cycle assessment and techno-economic analysis will also be necessary to quantify the environmental benefits and industrial competitiveness of CP fiber-based composites compared with conventional reinforcement systems. Such developments would strengthen the contribution of *Carica papaya* fiber composites to the transition toward a circular bioeconomy and more sustainable engineering materials.

## Figures and Tables

**Table 1:** Composition and designation of the unsaturated polyester (UP) composites reinforced with CP fibers extracted using different methods.

Sample Code	Fiber Extraction Method	Fiber Weight Fraction (wt.%)	UP Weight Fraction (wt.%)
UP	Neat unsaturated polyester resin	0	100
CPFRUP	Water-retted CP fibers	40	60
CPF5UP	CP fibers treated with 5 wt.% NaOH	46	54
CPF10UP	CP fibers treated with 10 wt.% NaOH	41	59

**Table 2:** Main characteristics of CP fibers used for composite manufacturing.

Property	CPFR	CPF5	CPF10
Extraction yield (%)	13.10	11.78	10.79
Average diameter (µm)	260 ± 60	180 ± 30	220 ± 50
Average length after carding (mm)	67.78 ± 21	72.59 ± 23	67.15 ± 27
Mat areal density (g/m^2^)	501.37	598.39	529.24
Density (g/cm^3^)	0.72 ± 0.05	1.06 ± 0.04	1.07 ± 0.03
Moisture content (%)	9.0 ± 0.3	9.1 ± 0.4	9.3 ± 0.4
Water absorption at 24 h (%)	225.0 ± 4.3	75.1 ± 16.8	47.5 ± 5.7
Tensile strength (MPa)	116.9 ± 67.7	277.0 ± 97.5	218.0 ± 132.5
Young’s modulus (GPa)	2.69 ± 1.17	4.52 ± 2.49	4.20 ± 2.07
Elongation at break (%)	0.90 ± 0.45	1.61 ± 0.62	1.55 ± 0.75

**Table 3:** Tensile, flexural, and Shore D hardness properties of polyester composites reinforced with CP fibers.

Composite	Shore D Hardness (HD)	Young’s Modulus (GPa)	Tensile Strength (MPa)	Elongation at Break (%)	Flexural Modulus (MPa)	Flexural Strength (MPa)	Flexural Strain at Break (%)
UP	77.2 ± 1.0 ^a^	0.57 ± 0.16 ^b^	21.56 ± 2.49 ^cd^	6.82 ± 1.43 ^a^	744 ± 63 ^c^	35.51 ± 1.48 ^b^	5.43 ± 0.31 ^a^
CPFRUP_MD	77.9 ± 3.2 ^a^	1.45 ± 0.32 ^a^	36.73 ± 7.02 ^b^	3.05 ± 0.74 ^bd^	2832 ± 536 ^b^	41.01 ± 8.55 ^b^	1.88 ± 0.18 ^c^
CPFRUP_CD	–	1.51 ± 0.17 ^a^	17.67 ± 3.04 ^c^	1.72 ± 0.35 ^cd^	2263 ± 291 ^b^	35.06 ± 6.92 ^b^	1.97 ± 0.23 ^bc^
CPF5UP_MD	66.1 ± 3.9 ^c^	1.85 ± 0.53 ^a^	55.29 ± 9.80 ^a^	4.13 ± 0.71 ^b^	4135 ± 607 ^a^	60.52 ± 7.26 ^a^	2.53 ± 0.27 ^bc^
CPF5UP_CD	–	1.92 ± 0.09 ^a^	24.20 ± 3.59 ^cd^	1.38 ± 0.55 ^c^	2530 ± 1306 ^b^	34.93 ± 11.26 ^b^	2.55 ± 0.51 ^bc^
CPF10UP_MD	70.5 ± 3.8 ^b^	2.05 ± 0.53 ^a^	35.55 ± 4.85 ^b^	3.04 ± 0.72 ^bd^	3361 ± 479 ^ab^	58.70 ± 11.97 ^a^	2.79 ± 0.90 ^b^
CPF10UP_CD	–	1.60 ± 0.32 ^a^	31.74 ± 8.22 ^bd^	2.03 ± 0.28 ^cd^	2438 ± 456 ^b^	35.28 ± 7.63 ^b^	2.13 ± 0.44 ^bc^

Values are reported as mean ± standard deviation (n = 6). Different letters within the same column indicate statistically significant differences according to Tukey’s HSD test (*p* < 0.05). Hardness measurements were performed independently of fiber orientation.

**Table 4:** Relative improvement of the mechanical properties with respect to neat polyester (UP).

Composite	Δ Tensile Strength (%)	Δ Young’s Modulus (%)	Δ Flexural Strength (%)	Δ Flexural Modulus (%)
CPFRUP_MD	+70.4	+154	+15.5	+281
CPFRUP_CD	−18.0	+165	−1.3	+204
CPF5UP_MD	+156.4	+225	+70.4	+456
CPF5UP_CD	+12.2	+237	−1.6	+240
CPF10UP_MD	+64.9	+260	+65.3	+352
CPF10UP_CD	+47.2	+181	−0.6	+228

**Table 5:** Low-velocity impact parameters of the composites (incident energy = 4.17 J).

Composite	Peak Force (N)	Maximum Deflection (mm)	Residual Deflection (mm)	Absorbed Energy (J)	Impact Stiffness (N/mm)
CPFRUP	184	14.9	12.3	2.21	12.3
CPF5UP	255	8.5	1.1	2.45	29.8
CPF10UP	344	5.9	0.6	2.37	58.6

**Table 6:** Benchmark of the Mechanical Properties of Natural Fiber Nonwoven Composites for Automotive Applications.

Composite	Matrix	Porosity (%)	Tensile Strength (MPa)	Tensile Modulus (GPa)	Flexural Strength (MPa)	Flexural Modulus (GPa)	Thermal Stability (°C)	References
CPF5UP (MD)	Polyester	~23	**55.3**	1.85	60.5	4.14	~240	This study
CPF10UP (MD)	Polyester	~13	35.6	2.05	58.7	3.36	~240	This study
CPFRUP (MD)	Polyester	~16	36.7	1.45	41.0	2.83	~240	This study
Neat UP resin	Polyester	0	21.6	0.57	35.5	0.74	60–100 ^1^	This study
PLA/TC (T2, CD)	PLA	40–61	24.4	2.88	44.8	3.91	239	[5]
PLA/flax (T2, CD)	PLA	41–62	27.1	3.27	61.2	4.03	239	[5]
PLA/hemp (T2, CD)	PLA	43–61	26.3	3.19	58.8	4.40	239	[5]
PP/date palm (core)	PP	24.7	21.1	2.93	34.7	2.44	–	[36]
PP/date palm (skin)	PP	22.1	20.7	2.23	36.8	2.70	–	[36]
PP/sisal	PP	36.9	23.5	2.93	38.2	2.44	–	[36]
PP/abaca	PP	40.3	32.1	2.70	40.8	2.85	–	[36]
Flax/PP (nonwoven)	PP	2–25 ^2^	–	–	–	2.6–4.4	~200	[42]
Flax/thermoset composite	Epoxy/Polyester	0–10	–	–	–	100–150	~200	[42]
FlexForm 50PP/50NF	PP	~30	24–27	~3	–	2.6	–	[36,40]

^1^ Heat deflection temperature (HDT) at 1.8 MPa for polyester resin. ^2^ Depending on the process (2–8% for compression molding, up to 25% for injection molding).

**Table 7:** Comparison between the developed composites and materials commonly used for automotive interior door panels.

Material	Typical Automotive Use	Advantages	Limitations	Comparison with Present Work
PP + Kenaf	Door panels	Lightweight	Thermoplastic processing	Similar stiffness
PP + Hemp	Door panels	High stiffness	Higher cost	Comparable application
PP + Wood fiber	Interior trim	Low cost	Moisture sensitivity	Similar sustainability
Glass fiber/PP	Door modules	High strength	High density	Present work is lighter and bio-based
ABS	Interior trim	Good appearance	Petroleum-based	Present work is renewable
CPF5UP	Proposed	High tensile/flexural performance	Thermoset	Suitable for semi-structural interiors
CPF10UP	Proposed	Better impact and moisture resistance	Slightly lower strength	Suitable for humid service environments

## Data Availability

Data are contained and presented within the article.
